# Assessing the impact of headaches and the outcomes of treatment: A systematic review of patient-reported outcome measures (PROMs)

**DOI:** 10.1177/0333102417731348

**Published:** 2017-09-18

**Authors:** Kirstie L Haywood, Tom S Mars, Rachel Potter, Shilpa Patel, Manjit Matharu, Martin Underwood

**Affiliations:** 1 Warwick Research in Nursing, Department of Health Sciences, Warwick Medical School, The University of Warwick, Gibbet Hill, Coventry, UK; 2 On behalf of the CHESS team; Warwick Clinical Trials Unit, Warwick Medical School, The University of Warwick, Gibbet Hill, Coventry, UK; 3 Warwick Clinical Trials Unit, Warwick Medical School, The University of Warwick, Gibbet Hill, Coventry, UK; 4 Headache Group, UCL Institute of Neurology, Queen Square, London, UK

**Keywords:** Headache, patient-reported outcome, validity, reliability, systematic review

## Abstract

**Aims:**

To critically appraise, compare and synthesise the quality and acceptability of multi-item patient reported outcome measures for adults with chronic or episodic headache.

**Methods:**

Systematic literature searches of major databases (1980–2016) to identify published evidence of PROM measurement and practical properties. Data on study quality (COSMIN), measurement and practical properties per measure were extracted and assessed against accepted standards to inform an evidence synthesis.

**Results:**

From 10,903 reviewed abstracts, 103 articles were assessed in full; 46 provided evidence for 23 PROMs: Eleven specific to the health-related impact of migraine (n = 5) or headache (n = 6); six assessed migraine-specific treatment response/satisfaction; six were generic measures. Evidence for measurement validity and score interpretation was strongest for two measures of impact, Migraine-Specific Quality of Life Questionnaire (MSQ v2.1) and Headache Impact Test 6-item (HIT-6), and one of treatment response, the Patient Perception of Migraine Questionnaire (PPMQ-R). Evidence of reliability was limited, but acceptable for the HIT-6. Responsiveness was rarely evaluated. Evidence for the remaining measures was limited. Patient involvement was limited and poorly reported.

**Conclusion:**

While evidence is limited, three measures have acceptable evidence of reliability and validity: HIT-6, MSQ v2.1 and PPMQ-R. Only the HIT-6 has acceptable evidence supporting its completion by all “headache” populations.

## Background

Headache disorders are common in the adult population; the most common – tension-type and migraine – have a one-year prevalence of 40% and 11% respectively (1,2,3). Between 2–4% of the general population experience chronic headache (4,5). Headache disorders can profoundly impact an individual’s functional ability and quality of life (3,6). Affecting primarily young adults, the personal and economic burden of headache is substantial and comparable to other chronic conditions such as congestive heart failure, hypertension, or diabetes (7).

An individual’s self-report of the presence, severity, frequency, and impact of headache is crucial to understanding the effectiveness of therapeutic interventions. Patient-reported outcome measures (PROMs), which seek to provide a patient-based assessment of the impact of headache on how people feel, function and live their lives, are now available. While recommendations to include PROMs in headache clinical trials are available (8,9), specific guidance for PROM-based outcome reporting does not exist. The integrity of PROM-based reporting is underpinned by clear evidence of essential measurement and practical properties in the clinical population of interest (10,11). It cannot be assumed that the reliability and validity of measure is consistent across different types of headache, and evidence of PROM performance across different sub-types is often not available (12). PROM score interpretation also requires guidance for what change in score reflects a meaningful change in “headache” for the individual patient (minimal important change (MIC)) and what difference reflects a meaningful difference between groups of patients defined by some external anchor (minimal important difference (MID)) (10,11). Structured reviews of PROM performance provide essential evidence to inform the selection of robust, relevant, and acceptable measures.

In this systematic review, we critically appraise, compare and synthesise published evidence of essential measurement and practical properties for clearly defined PROMs evaluated in adult headache populations. The review provides a transparent summary of the evidence base with which to inform PROM selection for future application in headache-specific research.

## Methods

### Identification of studies and PROMs: Search strategy

The search strategy was developed by experienced reviewers (KH, TM, RP, SP) and with expert librarian support to retrieve references relating to the development and/or evaluation of multi-item PROMs used in the assessment of adults (aged 18 years and above) with chronic or episodic headache including migraine.

Medical subject headings (MeSH terms) and free text searching were used to reflect three characteristics: a) population – headache and migraine; b) type of assessment – patient-reported outcome measures (PROMs); and c) measurement and practical properties (11,13,14). The full search strategy is available in Appendix 1.1.

**Figure 1.**
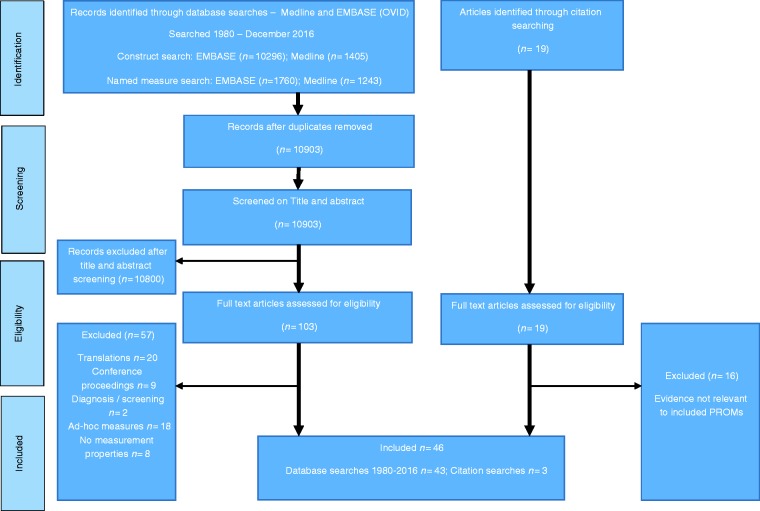
Review of measures used with people with headache – PRISMA flow diagram for article selection (search conducted 1980 to December 2016).

Two databases were searched (MEDLINE (OVID), EMBASE (OVID); 1980 to December 2016) (Figure 1). A subsequent search incorporated the names of more than 50 multi- and single-item measures identified during the initial search (Appendix 1.2 and 1.3). From a total of 39 multi-item PROMs thus identified, 16 had been superseded by revised measures or were no longer in use, as evidenced by their lack of inclusion in studies published post 2000 (Appendix 2). Given that such measures are unlikely to be of interest, the eligibility criteria for the review and analysis was revised to focus on PROMs in use post-2000.

The citation lists of included articles and existing reviews were also reviewed (15,16). Named author searches were conducted.

### Inclusion/exclusion criteria

Titles and abstracts of all articles were independently assessed for inclusion/exclusion by two reviewers (TM, KH) and agreement checked. Published articles were included if they provided evidence of development/evaluation for clearly defined, reproducible, multi-item PROMs, following self-completion by adults who self-reported or had been diagnosed by a clinician as having a headache disorder. Articles relating solely to the application of measures without some evidence of measurement and/or practical properties were excluded. Articles describing the translation of PROMs and/or evaluations in non-English speaking populations were also excluded. Conference papers and abstracts were excluded.

Included PROMs had to be in use in research published between 2000–2016. PROMs were categorised as: Generic (profile; utility) or condition-specific (headache; migraine). Clinician-reported, diagnostic and screening measures were excluded. Domain-specific measures that were not specific to the impact of headache, and measures that were not clearly reproducible, were excluded.

### Data extraction and appraisal

A data extraction form was informed by guidance for PROM evaluation (10,11,17), published PROM reviews (14,18,19) and the COnsensus-based Standards for the selection of health Measurement Instruments (COSMIN) checklist (20,21). The form captured both study and PROM-specific information. Population diagnosis and diagnostic criteria (if any) were extracted. We sought evidence on: Reliability (internal consistency; test–retest, intra/inter-tester); validity (content; construct; known groups); responsiveness; interpretation (minimal important change (MIC) and/or difference (MID)); and precision (data quality; end effects). Evidence for the practical properties included acceptability (relevance; respondent burden) and feasibility. Evidence of active patient involvement in PROM evaluation was also sought (18,22,23). All publications were double-assessed (KH, TM) and agreement checked.

### Assessment of study methodological quality

One experienced reviewer (KH) applied the COSMIN checklist to assess the methodological quality of included studies (20,21). Methodological quality was evaluated per measurement property on a four-point rating scale (excellent, good, fair, poor) and determined by the lowest rating of any items in each checklist section (21).

### Assessment of PROM quality

A similar checklist for PROM quality does not exist. Therefore, a pragmatic checklist informed by a synthesis of various recommendations was adopted (18,19,21,24) (Appendix 3: Table 2). To provide a global overview of the concepts captured within the reviewed headache-specific measures, items were categorised as per the domains of one of the most frequently used conceptual models of health-related quality of life (HRQOL) – the Ferrans revision to the Wilson and Cleary model (25,26).

### Data synthesis

A qualitative synthesis of evidence per reviewed PROM per reported measurement property informed the overall judgement of quality and acceptability. The synthesis combined four factors: a) study methodological quality (COSMIN scores); b) number of studies reporting evidence per PROM; c) results per measurement property (Appendix 3: Table 2); and d) evidence of consistency between evaluations (23,27). Two elements of the data synthesis are described: First, the overall quality of a measurement property was reported as adequate (+), conflicting (±), inadequate (−), or indeterminate (?). Second, evidence for the overall quality of evidence was categorised as “strong”, “moderate”, “limited”, “conflicting”, or “unknown” (27).

## Results

### Identification of studies and PROMs

Study and PROM identification is summarised per PRISMA guidance in Figure 1 (www.prisma-statement.org). Forty-six articles provided evaluative evidence for 23 PROMs (Appendices 4 and 5 (Tables 3 and 4)). Six assessed impact of headaches overall: The EUROLIGHT (28); Headache Activities of Daily Living Index (HADLI) (29); Headache-specific Disability Questionnaire (HDQ) (30); the Headache Impact Test (HIT) (3) and its short-form HIT-6 (31); and a headache-specific modification of the Short-Form 36-item Health Survey (32). Five were specific to the impact of migraine: Functional Assessment in Migraine questionnaire (FAIM) (33); Headache Needs Assessment Survey (HANA) (34); MIgraine Disability ASessment (MIDAS) (35); Migraine-Specific Quality of Life Questionnaire (MSQ v2.1) (36); and the Migraine-Specific Quality of Life (MSQOL) measure (37). Six assessed response to and/or satisfaction with migraine-specific drug treatment: Completeness of Response to migraine therapy (CORS) (38); Migraine Assessment of Current Therapy (Migraine-ACT) (39); Migraine-Treatment Assessment Questionnaire (M-TAQ) (40); Migraine-Treatment Optimisation Questionnaire (M-TOQ) (41); Migraine Treatment Satisfaction Measure (MTSM) (42); and the Patient Perception of Migraine Questionnaire – Revised (PPMQ-R) (43). Item content of all specific measures is illustrated in Appendix 6 (Table 5).

Finally, six generic measures had been assessed in headache populations: The Short-Form 36-item Health Survey (SF-36) (44), SF-12 (45), SF-8 (46), EuroQoL EQ-5D 3L (47), Health Utility Index-3 (HUI-3) (48) and the Quality of Well-being Scale (QWB) (49,50).

### Patient and study characteristics (Appendix 5 (Table 4))

Patient populations ranged from 18 to 83 years, were largely white, often with large proportions of female participants. Sample sizes ranged from 25 to more than 8,500. Populations included mixed, chronic, and/or episodic headache or migraine. Where clinician-based diagnosis was described, most adopted the International Classification of Headache Disorders (ICHD-II), available at http://www.ihs-klassifikation.de/en/. However, for many, patients were self-diagnosed, and a wide range of diagnostic criteria were described. Most studies were cross-sectional or longitudinal surveys. Nine were clinical trials or involving data secondary analysis. Fourteen studies were specific to PROM development and/or initial evaluations. Most evaluations were with US populations.

### Measurement properties and methodological quality

Study methodological quality per measurement property per reviewed PROM is presented in Appendix 7 (Table 6). The overall evidence synthesis is presented in Table 1.

**Table 1.** Data synthesis, levels of evidence and overall quality of reviewed PROMs in the headache population (n = 23)^a^. Note: CHESS PROMs Review – edited 27/07/17.

		Reliability			Validity		Construct validity		Responsiveness	
PROM^b^/study	Number of evaluations	Internal consistency	Temporal stability	Measurement error	Content validity	Structural validity	Hypothesis testing	Known groups	Responsiveness	Interpretation
Condition-specific (17)										
Migraine impact (5/17)										
FAIM (33)	1	+ Moderate			? Limited	+ Moderate	+ Moderate			
HANA (34)	1	+ Unknown	+ Unknown		? Unknown	? Unknown	? Unknown	? Unknown	ES only	
MIDAS (35)	13	+ Unknown	+ Unknown				+ Moderate	+ Moderate		
MSQ v2.1 (36)	11	+ Strong	± Conflicting		+ Limited	+ Strong	+ Moderate	+ Moderate		+ Limited
MSQoL (37)	3	+ Limited			+ Strong	+ Moderate	? Unknown	? Unknown		? Unknown
Headache-impact (6/11)										
EUROLIGHT (28)	1	? Unknown	? Unknown		? Limited		? Unknown	? Unknown		
HADLI (29)	1	+ Limited			? Unknown	+ Limited				
HDQ (30)	1	+ Limited			? Unknown	+ Limited				
HIT (3,57)	3		+ Moderate		+ Limited	+ Moderate	+ Moderate	+ Moderate		
HIT-6 (31)	13	+ Strong	+ Moderate		+ Limited	+ Moderate	+ Strong	+ Strong		+ Limited
SF-36 ‘Headache’ Modification (32)	1				? Unknown		? Unknown			
Response to migraine-specific treatment (6/17)										
CORS (38)	1	+ Limited			+ Moderate	+ Limited	? Limited		+ Limited	
M-ACT (39)	2		+ Moderate		? Unknown		? Unknown			
M-TAQ (40)	1		+ Limited		+ Limited		+ Limited			
M-TOQ (41)	1	+ Limited	+ Limited		+ Limited	+ Limited	+ Limited			
MTSM (42)	2	+ Limited			+ Moderate	+ Limited	+ Moderate	+ Moderate		
PPMQ-R (43)	2	+ Strong	+ Limited		+ Strong	+ Strong	+ Strong	+ Strong		+ Limited
Generic measures (6)										
Profile measures (3/6)										
SF-36 (44)	5						+ Moderate			
SF-12 (45)	1							+ Limited		
SF-8 (46)	4						+ Moderate	+ Moderate		
Utility measures (3/6)										
EuroQoL EQ-5D-3L (47)	3						+ Limited	+ Limited		
HUI-3 (48)	1							? Unknown		
QWB/QWB-SA (49,50)	1		± Conflicting					? Unknown		

a Data synthesis: The data were qualitatively synthesised to determine the overall quality of measurement properties and acceptability of each reviewed PROM. The synthesis took the following factors into account: a) methodological quality of the reviewed studies (COSMIN scores); b) the number of studies reporting evidence of measurement properties per PROM; c) the results for each measurement property for each PROM; and d) the consistency of results between reviewed studies.

The data synthesis score has two elements (19,27): First, the overall quality of a measurement property was reported as: adequate (+), not adequate (−), conflicting (±), or unclear/indeterminate (?) (see Table 1 for detail). Second, levels of evidence for the overall quality of each measurement property were further defined to indicate ‘strong’ – consistent findings in multiple studies of good methodological quality OR in one study of excellent quality; ‘moderate’ – consistent findings in multiple studies of fair methodological quality OR in one study of good methodological quality; ‘limited’ – one study of fair methodological quality; ‘conflicting’ – conflicting findings; or ‘unknown’ evidence – only studies of poor methodological quality. Where the data entry box is left blank, this signifies no available evidence.

*PROMs assessing migraine and headache-specific impact (n = 11)*

Apart from the FAIM, MSQ v2.1, MSQoL and HIT, all measures lack a clear description of aim, the concepts being measured, or the process of item generation. The FAIM (33), MSQ v2.1 (36) and MSQoL (37) involved expert clinicians and patients in item generation, supporting a positive rating of content validity.

The HIT “item bank” was informed by four legacy measures – the MIDAS, MSQ (v1.0), Headache Disability Index (HDI) and Headache Impact Questionnaire (HIMQ) – and consultation with clinicians (3). Apart from the MSQ, item generation for these measures is poorly reported but largely driven by clinical opinion. Additional evaluations of the content validity of the item bank or short form measures is not described. Clinical opinion, literature review, and/or the completion of established questionnaires were the main sources of items for the remaining measures. There was no evidence of active patient collaboration in PROM development and/or evaluation.

The shortest measures are the MIDAS (five items) and HIT-6 (six items); the longest is the 103-item EUROLIGHT (Table 2). Apart from the FAIM, all assess headache/migraine symptomology. While five headache-specific measures assess pain, the migraine-specific measures do not. Only the HANA, MSQv2.1 and HIT-6 assess fatigue.

All assess the impact of headache/migraine on social function, activities of daily living and/or work. Seven – FAIM, HANA, MSQv2.1, MSQOL, HIT, HIT-6, and EUROLIGHT – assess the emotional burden of headache/migraine; five of these – FAIM, MSQv2.1, HIT, HIT-6, and EUROLIGHT – plus the HADLI, assess the impact on cognition and difficulty with thinking.

Acceptable evidence of measurement dimensionality from studies of at least moderate methodological quality was reviewed for five measures – FAIM (33), MSQv2.1 (12,51), MSQoL (52), HIT (3), HIT-6 (53); three have moderate to strong evidence of both structural validity *and* internal consistency – FAIM (33), MSQ v2.1 (12,36,51,54) and the HIT-6 (31,41,53,55,56) (Table 1; Appendix 7). Three measures have acceptable evidence of the reliability of internal consistency from studies of at least moderate methodological quality, supporting application in the assessment of groups (FAIM) (33) and individuals (MSQ v2.1 [12,36,51], HIT-6 [53,56]) (Table 1; Appendix 7); however, for the majority, evidence was limited (n = 3), from poor quality studies (n = 3) or not available (n = 1). Only the HIT (31,57) and HIT-6 (31,53,56,57) have acceptable evidence of temporal stability supporting application in the assessment of groups and individuals. Evidence for the remaining measures was limited.

Five measures have acceptable evidence from good quality studies describing their construct validity – FAIM (33), MIDAS (56), MSQ v2.1 (12,36,43,53), HIT (57) and HIT-6 (12,53,56,57). For the remaining measures, evidence was of poor quality (n = 4) or not available (n = 2); authors often failed to hypothesise *a priori* the association between variables.

Evidence of responsiveness was limited. Statistically significant between-group differences for average HIT-6 and total HIT change scores were reported for patients categorised by self-reported change (better/same/worse) in physical activity, level of frustration or daily activities following a three-month follow-up period of “usual care” (31).

Large and moderate effect size statistics were reported for the MSQv2.1 (12) and HIT-6 (53) in patients who reported large or moderate improvement in the number of headache days following a pharmaceutical-based clinical trial, respectively. Following a non-comparative, observational study of zolmitriptan for an acute migraine attack, small and moderate ES statistics were reported for the SF-36 and MSQoL respectively (52).

Following completion of the HIT-6 by patients with chronic daily headache in a trial of usual medical care (UMC) versus UMC plus acupuncture, an anchor-based estimate of the MIC was calculated as approximately 3.7; the MID was estimated as 2.3 (58). Change in HIT-6 scores that exceeded the proposed MIC were reported in patients with chronic migraine receiving onabotulinumtoxinA in a placebo-controlled double blind trial; a between-group difference that exceeded the MID, in favour of the active treatment, was also reported (59).

Both anchor-based (60,61) and distribution-based estimates (60) were calculated for the MSQv2.1 following completion by patients with chronic migraine. Cole et al. (60) proposed an MIC of 5.0 for the RR domain, with ranges for the RP (5.0 to 7.9) and EF (range 8.0 to 10.6) domains; MIDs were recommended as RR 3.2, RP 4.6, EF 7.5 (60). A between-group difference that exceeded the proposed MID, in favour of the active treatment, was reported for the MSQv2.1 RR domain only in patients with chronic migraine receiving onabotulinumtoxinA in a placebo-controlled double blind trial (59). However, within-individual change scores were larger than the proposed MIC for each domain for patients receiving active treatment.

*PROMs assessing response to or satisfaction with migraine-specific treatment (six measures)*

Four of the six measures – the CORS, M-TOQ, MTSM and PPMQ-R – have acceptable descriptions of the measurement aim, conceptual underpinning and item generation. Although detail is limited, three measures – CORS, MTSM and PPMQ-R – involved both expert clinicians and patients in item generation (the MTSM involved US and UK participants), supporting a positive rating of content validity; the M-TAQ utilised patient interviews and focus groups, with additional reference to established treatment optimisation measures.

Item generation for the M-ACT (39) and the M-TOQ (41) was informed by clinical evidence and the consensus of clinical headache experts and researchers; patients were not involved, supporting a negative rating of content validity. There was no evidence of active patient collaboration.

The shortest measures are the M-ACT (four items) and M-TOQ-5 (five items); the longest is the 45-item MTSM (Appendix 4). Apart from the M-ACT and M-TAQ, all assess migraine symptomology, including pain severity, and the wider impact on activities of daily living and/or work; the PPMQ-R also assesses limitations in social functions (Appendix 6). The CORS, M-TOQ-15 and PPMQ-R assess the emotional burden of migraine; only the CORS and PPMQ-R also assess cognition and difficulty with thinking. Three measures assess if the patient has “returned to normal” – CORS, M-ACT, and M-TOQ. All assess confidence in/or satisfaction with treatment; the M-TOQ assesses treatment side-effects.

Only the PPMQ-R has acceptable evidence of measurement dimensionality *and* internal consistency reliability from studies of at least moderate methodological quality (Table 1; Appendix 7). For three measures – CORS, M-TOQ, and MTSM – evidence was acceptable but limited.

Only the M-ACT has acceptable evidence of temporal stability from several studies of fair methodological quality, supporting application in the assessment of groups (Table 1; Appendix 7). Evidence for three measures – M-TAQ, M-TOQ, and PPMQ-R – was limited to single studies judged to be of fair quality (Table 1; Appendix 7). Only the PPMQ-R and MTSM have acceptable evidence of construct validity from good quality studies. For the remaining measures, evidence was limited (CORS, M-TAQ, and M-TOQ) or from poor quality (M-ACT) studies.

Following a two-month pharmaceutical trial, small to moderate change score correlations between the CORS and the PPMQ-R supported *a priori* hypothesised associations, providing acceptable, but limited, evidence of responsiveness (38). Further criterion-based evidence, comparing the comparative CORS with change in CORS sub-sets at two months, provided additional, hypothesis-driven evidence of responsiveness (38). Small to moderate effect size statistics were reported for the PPMQ-R in patients categorised by self-reported improvement (range 0.14 to 0.50) or worsening (range 0.06 to 0.23) in pain severity; the largest ES were reported for the Efficacy and Function domains (43).

The Standard Error of Measurement (SEM) was calculated for the PPMQ-R, as a reflection of the within-individual minimal change in score (MIC) (43). Apart from the Cost domain (SEM 11.0), SEM estimates ranged 3.4 (Bothersome) to 5.4 (Total score), supporting an MIC recommendation of five points for the total score and Efficacy, Function and Ease of Use domains. Results suggest that the Cost domain is highly variable and not responsive to change in migraine severity or role limitation.

Estimates of the minimally important change and minimally important difference were reviewed for three headache-specific measures: MSQ v2.1 (36), HIT-6 (31), PPMQ-R (43). Completion of the HIT-6 by Dutch patients with chronic tension-type headache (62) and episodic migraine (63) suggested a wider range of MIC values, from −2.5 (63) to −8.0 (62) than that determined in a US population with chronic daily headache (−3.7) (58). The differences were largely explained by use of different anchors – where a greater perceived change was the imposed anchor, a larger MIC was calculated. An MIC of >8.0 suggests that improvement must be present in at least two of the six HIT-6 items (62), which may be judged a relevant treatment effect (62,63). Similarly, suggested MID values range from −1.5 (episodic migraine) (63) to −2.3 (chronic daily headache) (58).

*Generic PROMs (n = 6)*

Evaluations of all generic measures in the headache population were very limited. There was no evidence exploring the content validity or relevance of the six reviewed generic measures with the headache population. There was no evidence of active patient collaboration.

Where applicable, there was no evidence of measurement dimensionality or internal consistency reliability (Table 1). Just one measure – the QWB-SA – had conflicting evidence of temporal stability from one study, judged to be of poor methodological quality (64) (Table 1; Appendix 7).

Acceptable evidence of construct validity from several studies judged to be of fair or good methodological quality was reviewed for both the SF-36 (36,55,65) and the SF-8 (7,31,57,56); for the SF-12 evidence was limited (Table 1; Appendix 7). For the remaining measures, evidence was limited (EQ-5D) or of poor quality (HUI-3, QWB). There was no evidence of measurement responsiveness.

## Discussion

High quality, relevant and acceptable PROMs provide patient-derived evidence of the impact of headache and the relative benefit of associated healthcare at both the time of the headache and the intervening period. The importance of capturing the patient perspective is reflected in the large number of measures included in this review. However, apart from two condition-specific – HIT-6 and MSQv2.1 – and one treatment-response – PPMQ-R – measures, for which strong evidence was reviewed, evidence was largely limited or not available.

This is the first systematic review to include a methodological assessment of both study and PROM quality in the headache population. Clarity in PROM focus is an essential, but often overlooked aspect of PROM development (24,80). Except for four condition-specific (MSQ v2.1, MSQoL, HIT and HIT-6) and four treatment-response measures (CORS, M-TOQ, MTSM and PPMQ-R), all lacked a clear description of the measurement aim. Moreover, the condition-attribution of measures was not always self-evident: Just three ‘migraine-specific’ measures assessed the impact of “migraine” (FAIM, MSQ v2.1 and MSQoL). The HANA includes both “migraine” and “headache” in the item stem and, despite the name, the MIDAS assesses the impact of “headache”. It is suggested that the attribution of “headache” supports a “broader” assessment than would be achieved with “migraine”; moreover, many patients may be unaware of a migraine diagnosis (3). The HIT item content was informed by both migraine (MSQ and MIDAS) and headache-specific (HIMQ, HDI) measures; a content comparison failed to reveal any systematic differences in concept coverage, and further evaluation in a mixed population supported the uni-dimensionality of headache disability (3). Evidence further supports the ability of the HIT to assess headache disability across a wide spectrum of impact, avoiding the potential for ceiling effects, following completion by headache and migraine populations (3,63). Just four measures (the HIT-6, HADLI, HDQ and MIDAS) have been evaluated in both headache and migraine populations. However, while evidence is strong for the HIT-6, the remaining measures should be applied with caution.

Except for two condition-specific (MSQv2.1 and MSQoL) and four treatment-response measures (CORS, M-TAQ, MTSM and PPMQ-R), the extent of patient participation was limited and poorly detailed. Moreover, except for three measures (MSQoL, PPMQ-R and EUROLIGHT) PROM relevance, content and face validity was not explicitly explored with patients and/or expert panels. Item content for the remaining measures was informed by a mix of qualitative research with clinicians, reference to existing measures, published literature and/or completed questionnaires. Successful treatment for headache disorders should seek to improve both overall quality of life, as well as an individual’s quality of life during the attack (37); assessment should seek to capture these distinctions.

Although varying in length, there was a similarity of item content across condition-specific measures. Most assessed headache/migraine-related symptomology; pain severity was commonly assessed by headache-specific and treatment-response measures, but not by the migraine-specific measures. Just two measures (MSQv2.1 and HIT-6) assessed fatigue. Measures with a primary focus on symptomology have been criticised for failing to take into consideration the longer-term consequence of, or fear associated with, a potentially-severe headache or migraine, such as evading commitments or making plans (81,82). Nevertheless, except for the FAIM and HANA, all condition-specific and most treatment-response measures also assessed the wider impact of headache on social function and interactions, activities of daily living and/or work. Several measures (MSQv2.1, HIT, HIT-6, EUROLIGHT, CORS and PPMQ-R) also assessed both the emotional burden and cognitive impact of headache/migraine.

Three condition-specific (FAIM, MSQv2.1 and HIT-6) and one treatment-response (PPMQ-R) measures have strong evidence of both structural validity and reliability of internal consistency. Factor analysis supported the uni-dimensionality of the FAIM following completion by migraineurs, and the HIT-6 as a measure of “headache disability” following completion by mixed populations. The three-domain structure of the MSQv2.1 was supported – Role Restriction (RR), Role Prevention (RP) and Emotional Function (EF) – following completion in both chronic and episodic migraine populations. However, for most measures, evidence of structural validity or reliability of internal consistency was limited, from methodologically-poor quality studies, or not available. Evidence of temporal stability was also limited, and available only for the HIT, HIT-6, M-ACT, M-TAQ, M-TOQ and PPMQ-R. There was no evaluation of measurement error.

Five condition-specific (FAIM, MIDAS, MSQ v2.1, HIT and HIT-6), two treatment-response (MTSM and PPMQ-R) and two generic (SF-36 and SF-8) measures have acceptable evidence of construct validity from good quality studies. For the remaining measures, evidence was limited, of poor methodological quality, or not available. Methodological inadequacies included small sample sizes and a failure to hypothesise *a priori* the expected association between variables. As reported in other reviews (18,19), there was limited evidence of responsiveness: Just two studies (31,38) provided acceptable, but limited, evidence for the CORS and HIT measures. Evaluative measures require evidence of responsiveness to demonstrate that they can detect real change in condition over time; without such evidence, measures should be applied with caution.

While a limitation of the review is that we have only included evaluations in English, the context, setting and population are important in appraising evidence of PROM measurement and practical properties (83). Moreover, the diversity of reviewed measures reflects the wide range of assessment approaches in current use. Reviewed studies were of adults aged 18 years and over; with no upper age-limit imposed. All reviewed studies excluded people with significant co-morbidities. We are confident that the results are generalisable to the wider population of English-speaking adults with headache, but may not reflect the experience of adults with headache who have significant co-morbidities or do not speak English.

All data from included studies was double extracted and agreement checked (KH, TM). However, the COSMIN grading and synthesis score was applied by a single, experienced reviewer (KH). Although applied in several recent reviews (19,84), the grading system itself lacks robust evidence of reliability and validity and should therefore be interpreted with caution.

The lack of reporting guidance and significant heterogeneity in outcome assessment detailed in this review highlight the importance of establishing guidance on outcome reporting in this population. Future research should seek to establish international, multi-perspective guidance for a core set of outcomes to include in future headache research and across routine practice settings. The first step in this process is to seek consensus on which outcomes should be assessed, as a minimum, in future clinical trials or routine practice settings (85). Informed by recommendations from this review, the second step is to determine the “best way” to assess these core outcomes.

Although many PROMs were reviewed following their evaluation in the headache and/or migraine population, study methodological quality was often poor and evidence of essential measurement properties largely unavailable or limited. Such limitations hinder PROM data interpretation from clinical trials, audit, or quality assurance initiatives. However, three measures – HIT-6, MSQv2.1 and the PPMQ-R – had acceptable, and often strong, evidence of reliability and validity following completion by patients with headache (HIT-6) or migraine (HIT-6, MSQv2.1, PPMQ-R), and are recommended for consideration in future clinical research and routine practice settings as measures of headache-specific impact, migraine-specific impact, or migraine-treatment response respectively. However, the similarity of item content across all three measures suggests that a further exploration of the attribution, relevance and acceptability of the measures with representative members of the patient population is warranted. Further comparative evidence of widely-used generic measures and evidence of measurement responsiveness of all measures is urgently required.

## Supplementary Material

Supplementary Material

## Article highlights

- Despite the large number of reviewed PROMs currently used with patients with headache, most have not involved patients in the development process and may lack relevance to the patients’ experience of headache. Most also lack clarity with regard to measurement aim and have limited evidence of essential measurement properties, limiting confidence in data interpretation. These PROMs should be used and interpreted with caution.
- Strong evidence of reliability and validity was reviewed for three measures, HIT-6, MSQv2.1 and the PPMQ-R, supporting recommendation for consideration in future clinical research or routine practice settings. However, unlike the MSQv2.1 and PPMQ-R, patients were not involved in item generation for the HIT-6.
- The review has highlighted significant heterogeneity in outcome reporting in headache studies, raising concerns over reporting bias and limiting the conduct of systematic reviews and meta-analyses of evidence. International multi-perspective consensus on the most important outcomes – both which outcomes and how to assess them – is required, and can be supported by the findings from this review.
