# Supplementary material for: Assessing the impact of headaches and the outcomes of treatment: A systematic review of patient-reported outcome measures (PROMs)
Source: Cephalalgia. 2017 Sep 18;38(7):1374–86. doi: 10.1177/0333102417731348 (PMC6024352; doi:10.1177/0333102417731348)
Supplement: Supplementary Material [file supplementary_material.pdf]

Headache Review – Cephalalgia

Appendix 1.1 : Search strategies: Construct searches

Database: Embase Classic+Embase <1947 to 2017 Week 16>

Search Strategy:

----

1 exp chronic daily headache/ or exp episodic tension headache/ or exp headache/ or exp primary headache/ or exp chronic tension headache/ or exp new daily persistent headache/ or exp secondary headache/ or exp tension headache/ (201706)

2 (headache* or migraine*).ti,ab. (136921)

3 (headache* adj3 (mixed or combination or tension or tension type or muscle contraction or psychomyogenic or stress or ordinary or essential or psychogenic)).tw. (6527)

4 ((chronic adj2 daily adj2 headache*) or (daily adj2 persistent adj2 headache*)).ti,ab. (1530)

5 long term headache*.tw. (43)

6 1 or 2 or 3 or 4 or 5 (244190)

7 (daily or persistent or chronic).mp. (2456082)

8 6 and 7 (56510)

9 exp migraine aura/ or exp ophthalmoplegic migraine/ or exp migraine/ or exp migraine with aura/ or exp migraine without aura/ (57058)

10 ((withdrawal or overuse or "over use" or "over-use" or misuse or "mis-use" or abuse or induced) adj5 (medication* or medicine* or analges* or drug* or opiate* or opioid* or nsaids or non-opiate* or non opiate or ergot* or painkiller* or pain killer* or pain-killer*) adj5 (headache* or migraine*)).mp. (20637)

11 ((rebound or transformed) adj5 (headache* or migraine*)).ti,ab. (450)

12 8 or 9 or 10 or 11 (119498)

13 (HR-PRO or HRPRO or HRPRO or HRQL or HRQoL or QL or QoL).mp. (73860)

14 (quality of life or life quality).tw. (310350)

15 (health index or health indices or health profile).mp. (5057)

16 (patient or self or child or parent or carer or proxy).mp. (8459912)

17 (report or reported or reporting or rated or rating or ratings or based or assessed or assessment assessments or disability or function or functional or functions or subjective or utility or utilities or wellbeing or well being).mp. (12222752)

18 (index or indices or instrument or instruments or measure or measures or questionnaire or questionnaires or profile or profiles or scale or scales or score or scores or status or survey or surveys).ti,ab. (5157109)

19 health related quality of life.ti,ab. (44869)

20 quality adjusted life year.ti,ab. (5178)

21 QALY.tw. (11466)

22 value of life.tw. (309)

23 ((health adj2 utility*) or disutili*).mp. (2799)

24 willingness to pay.tw. (5728)

25 contingent valuation.tw. (697)

26 standard gamble.tw. (945)

27 SG.tw. (11070)

28 time tradeoff.tw. (250)

29 time trade off.tw. (1369)

30 TTO.tw. (1299)

31 mapping.tw. (161946)

32 cross walking.tw. (17)

33 transfer to utility.tw. (12)

34 13 or 14 or 15 or 16 or 17 or 18 or 19 or 20 or 21 or 22 or 23 or 24 or 25 or 26 or 27 or 28 or 29 or 30 or 31 or 32 or 33 (17788699)

35 exp 'intermethod comparison'/ or exp 'data collection method'/ or exp 'validation study'/ or exp 'feasibility study'/ or exp 'pilot study'/ or exp 'psychometry'/ or exp 'reproducibility'/ or reproducib*.ab,ti. or 'audit'.ab,ti. or psychometr*.ab,ti. or clinimetr*.ab,ti. or clinometr*.ab,ti. or exp 'observer variation'/ or 'observer variation'.ab,ti. or exp 'discriminant analysis'/ or exp 'validity'/ or reliab*.ab,ti. or valid*.ab,ti. or 'coefficient'.ab,ti. or 'internal consistency'.ab,ti. or (cronbach* and ('alpha' or 'alphas')).ab,ti. or 'item correlation'.ab,ti. or 'item correlations'.ab,ti. or 'item selection'.ab,ti. or 'item selections'.ab,ti. or 'item reduction'.ab,ti. or 'item reductions'.ab,ti. or 'agreement'.ab,ti. or 'precision'.ab,ti. or 'imprecision'.ab,ti. or 'precise values'.ab,ti. or 'test-retest'.ab,ti. or ('test' and 'retest').ab,ti. or (reliab* and ('test' or 'retest')).ab,ti. or 'stability'.ab,ti. or 'interrater'.ab,ti. or 'inter-rater'.ab,ti. or 'intrarater'.ab,ti. or 'intra-rater'.ab,ti. or 'intertester'.ab,ti. or 'inter-tester'.ab,ti. or 'intratester'.ab,ti. or 'intra- tester'.ab,ti. or 'interobeserver'.ab,ti. or 'inter-observer'.ab,ti. or 'intraobserver'.ab,ti. or 'intra- observer'.ab,ti. or 'intertechnician'.ab,ti. or 'inter-technician'.ab,ti. or 'intratechnician'.ab,ti. or 'intra- technician'.ab,ti. or 'interexaminer'.ab,ti. or 'inter-examiner'.ab,ti. or 'intraexaminer'.ab,ti. or 'intra-examiner'.ab,ti. or 'interassay'.ab,ti. or 'inter-assay'.ab,ti. or 'intraassay'.ab,ti. or 'intra-assay'.ab,ti. or 'interindividual'.ab,ti. or 'inter-individual'.ab,ti. or 'intraindividual'.ab,ti. or 'intra-individual'.ab,ti. or 'interparticipant'.ab,ti. or 'inter-participant'.ab,ti. or 'intraparticipant'.ab,ti. or 'intra- participant'.ab,ti. or 'kappa'.ab,ti. or 'kappas'.ab,ti. or 'coefficient of variation'.ab,ti. or repeatab*.ab,ti. or ((replicab* or 'repeated') and ('measure' or 'measures' or 'findings' or 'result' or 'results' or 'test' or 'tests')).ab,ti. or generaliza*.ab,ti. or generalisa*.ab,ti. or 'concordance'.ab,ti. or ('intraclass' and correlation*).ab,ti. or 'discriminative'.ab,ti. or 'known group'.ab,ti. or 'factor analysis'.ab,ti. or 'factor analyses'.ab,ti. or 'factor structure'.ab,ti. or 'factor structures'.ab,ti. or

'dimensionality'.ab,ti. or subscale*.ab,ti. or 'multitrait scaling analysis'.ab,ti. or 'multitrait scaling analyses'.ab,ti. or 'item discriminant'.ab,ti. or 'interscale correlation'.ab,ti. or 'interscale correlations'.ab,ti. or (('error' or 'errors') and (measure* or correlat* or evaluat* or 'accuracy' or 'accurate' or 'precision' or 'mean')).ab,ti. or 'individual variability'.ab,ti. or 'interval variability'.ab,ti. or 'rate variability'.ab,ti. or 'variability analysis'.ab,ti. or ('uncertainty' and ('measurement' or 'measuring')).ab,ti. or 'standard error of measurement'.ab,ti. or sensitiv*.ab,ti. or responsive*.ab,ti. or ('limit' and 'detection').ab,ti. or 'minimal detectable concentration'.ab,ti. or interpretab*.ab,ti. or (small* and ('real' or 'detectable') and ('change' or 'difference')).ab,ti. or 'meaningful change'.ab,ti. or 'minimal important change'.ab,ti. or 'minimal important difference'.ab,ti. or 'minimally important change'.ab,ti. or 'minimally important difference'.ab,ti. or 'minimal detectable change'.ab,ti. or 'minimal detectable difference'.ab,ti. or 'minimally detectable change'.ab,ti. or 'minimally detectable difference'.ab,ti. or 'minimal real change'.ab,ti. or 'minimal real difference'.ab,ti. or 'minimally real change'.ab,ti. or 'minimally real difference'.ab,ti. or 'ceiling effect'.ab,ti. or 'floor effect'.ab,ti. or 'item response model'.ab,ti. or 'irt'.ab,ti. or 'rasch'.ab,ti. or 'differential item functioning'.ab,ti. or 'dif'.ab,ti. or 'computer adaptive testing'.ab,ti. or 'item bank'.ab,ti. or 'cross-cultural equivalence'.ab,ti. (5156030)

36 (addresses or biography or case reports or comment or directory or editorial or festschrift or interview or lectures or legal cases or legislation or letter or news or newspaper article or patient education handout or popular works or congresses or consensus development conference or consensus development conference, nih or practice guideline).pt. not (*animals/ not *humans/) (1526497)

37 (12 and 34 and 35) not 36 (20124)

38 limit 37 to (human and english language and (adult <18 to 64 years> or aged <65+ years>)) (10502)

39 limit 38 to yr="1980 - 2016" (10296)

Database: Ovid MEDLINE(R) <1946 to April Week 2 2017>

Search Strategy:

----

1 Headache/ (26018)

2 exp headache disorders/ or exp headache disorders, primary/ (32133)

3 exp Tension-Type Headache/ (1860)

4 (headache* adj3 (mixed or combination or tension or tension type or muscle contraction* or psychomyogenic or stress or ordinary or essential or psychogenic)).tw. (4133)

5 ((chronic adj2 daily adj2 headache*) or (daily adj2 persistent adj2 headache*)).ti,ab. (950)

6 (headache* or hemicrania simplex).mp. (74687)

7 long term headache*.mp. (19)

8 chronic headache*.mp. (1518)

9 exp Headache Disorders/ (32133)

10 tension headache*.mp. (1004)

11 1 or 2 or 3 or 4 or 5 or 6 or 7 or 8 or 9 or 10 (88376)

12 (daily or persistent or chronic).mp. (1583660)

13 11 and 12 (17326)

14 migraine*.mp. or exp Migraine with Aura/ or exp Migraine Disorders/ or exp Ophthalmoplegic Migraine/ or exp Migraine without Aura/ (32683)

15 (withdrawal or overuse or "over use" or "over-use" or misuse or "mis-use" or abuse or induced).mp. adj5 (medication* or medicine* or analges* or drug* or opiate* or opioid* or NSAIDS or non-opiate* or non opiate* or ergot* or painkiller* or pain killer* or pain-killer*).ti,ab. adj5 (headache* or migraine*).ti,ab. (3474)

16 ((rebound or transformed) adj5 (headache* or migraine*)).ti,ab. (327)

17 13 or 14 or 15 or 16 (45932)

18 (HR-PRO or HRPRO or HRPRO or HRQL or HRQoL or QL or QoL or PRO or PROs or PROM or PROMs).mp. (189311)

19 (quality of life or life quality).mp. (228883)

20 (health index* or health indices or health profile* or health status).mp. (128028)

21 ((patient or self or proxy) adj (appraisal* or appraised or report or reported or reporting or rated or rating or ratings or based or assessed or assessment*)).mp. (155466)

22 ((disability or function or functional or functions or subjective or utility or utilities or wellbeing or well being or health) adj2 (index or indices or instrument or instruments or measure or measures or questionnaire* or profile or profiles or scale or scales or score or scores or status or survey or surveys)).ti,ab. (182045)

23 health related quality of life.ti,ab. (27920)

24 quality adjusted life year.ti,ab. (3358)

25 QALY.tw. (5322)

26 value of life.tw. (249)

27 ((health adj2 utility*) or disutili*).mp. (1384)

28 willingness to pay.tw. (3086)

29 contingent valuation.tw. (498)

30 standard gamble.tw. (749)

31 SG.tw. (6571)

32 time tradeoff.tw. (252)

33 time trade off.tw. (884)

34 TTO.tw. (746)

35 mapping.tw. (122495)

36 cross walking.tw. (6)

37 transfer to utility.tw. (8)

38 18 or 19 or 20 or 21 or 22 or 23 or 24 or 25 or 26 or 27 or 28 or 29 or 30 or 31 or 32 or 33 or 34 or 35 or 36 or 37 (834011)

39 (instrumentation or methods).sh. or Validation Stud- ies.pt. or Comparative Study.pt. or exp Psychometrics/ or psychometr*.ti,ab. or clinimetr*.tw. or clino- metr*.tw. or exp "Outcome Assessment (Health Care)"/ or outcome assessment.ti,ab. or outcome measure*.tw. or exp observer variation/ or observer variation.ti,ab. or exp Health Status Indicators/ or exp reproducibility of results/ or reproducib*.ti,ab. or exp discriminant analysis/ or reliab*.ti,ab. or unreliab*.ti,ab. or valid*.ti,ab. or coefficient.ti,ab. or homogeneity.ti,ab. or homogeneous.ti,ab. or internal consistency.ti,ab. or (cronbach* and (alpha or alphas)).ti,ab. or (item and (correlation* or selection* or reduction*)).ti,ab. or agreement.ti,ab. or precision.ti,ab. or imprecision.ti,ab. or "precise values".ti,ab. or test- retest.ti,ab. or (test and retest).ti,ab. or (reliab* and (test or retest)).ti,ab. or stability.ti,ab. or interrater.ti,ab. or inter-rater.ti,ab. or intrarater.ti,ab. or intra-rater.ti,ab. or intertester.ti,ab. or inter-tester.ti,ab. or intratester.ti,ab. or intra-tester.ti,ab. or interobserver.ti,ab. or inter-observer.ti,ab. or intraobserver.ti,ab. or intra- observer.ti,ab. or intertechnician.ti,ab. or inter-techni- cian.ti,ab. or intratechnician.ti,ab. or intra-technician.ti,ab. or interexaminer.ti,ab. or inter-examiner.ti,ab. or intraex- aminer.ti,ab. or intra-examiner.ti,ab. or interassay.ti,ab. or inter-assay.ti,ab. or intraassay.ti,ab. or intra-assay.ti,ab. or interindividual.ti,ab. or inter-individual.ti,ab. or intraindi- vidual.ti,ab. or intra-individual.ti,ab. or interparticipant.ti,ab. or inter-participant.ti,ab. or intraparticipant.ti,ab. or intra-participant.ti,ab. or kappa.ti,ab. or kappa-s.ti,ab. or kappas.ti,ab. or repeatab*.ti,ab. or ((replicab* or repeated) and (measure or measures or findings or result or results or test or tests)).ti,ab. or generaliza*.ti,ab. or general- isa*.ti,ab. or concordance.ti,ab. or (intraclass and correlation*).ti,ab. or discriminative.ti,ab. or "known group".ti,ab. or factor analysis.ti,ab. or factor analyses.ti,ab. or dimension*.ti,ab. or subscale*.ti,ab. or (multitrait and scaling and (analysis or analyses)).ti,ab. or item discriminant.ti,ab. or interscale correlation*.ti,ab. or

error.ti,ab. or errors.ti,ab. or "individual variabil- ity".ti,ab. or (variability and (analysis or val- ues)).ti,ab. or (uncertainty and (measurement or measuring)).ti,ab. or "standard error of measure-ment".ti,ab. or sensitiv*.ti,ab. or responsive*.ti,ab. or ((minimal or minimally or clinical or clinically) and (important or significant or detectable) and (change or difference)).ti,ab. or (small* and (real or detectable) and (change or difference)).ti,ab. or meaningful change.ti,ab. or "ceiling effect".ti,ab. or "floor effect".ti,ab. or "Item response model".ti,ab. or IRT.ti,ab. or Rasch.ti,ab. or "Differential item functioning".ti,ab. or DIF.ti,ab. or "computer adaptive testing".ti,ab. or "item bank".ti,ab. or "cross-cultural equivalence".ti,ab. (5772794)

40 (addresses or biography or case reports or comment or directory or editorial or festschrift or interview or lectures or legal cases or legislation or letter or news or newspaper article or patient education handout or popular works or congresses or consensus development conference or consensus development conference, nih or practice guideline).pt. not (*animals/ not *humans/) (3636046)

41 (17 and 38 and 39) not 40 (2028)

42 limit 41 to (english language and humans and "all adult (19 plus years)") (1412)

43 limit 42 to yr="1980 - 2016" (1405)

Appendix 1.2 Named measure searches

Database: Embase Classic+Embase <1947 to 2017 Week 16>

Search Strategy:

----

1 exp chronic daily headache/ or exp episodic tension headache/ or exp headache/ or exp primary headache/ or exp chronic tension headache/ or exp new daily persistent headache/ or exp secondary headache/ or exp tension headache/ (201706)

2 (headache* or migraine*).ti,ab. (136921)

3 (headache* adj3 (mixed or combination or tension or tension type or muscle contraction or psychomyogenic or stress or ordinary or essential or psychogenic)).tw. (6527)

4 ((chronic adj2 daily adj2 headache*) or (daily adj2 persistent adj2 headache*)).ti,ab. (1530)

5 long term headache*.tw. (43)

6 1 or 2 or 3 or 4 or 5 (244190)

7 (daily or persistent or chronic).mp. (2456082)

8 6 and 7 (56510)

9 exp migraine aura/ or exp ophthalmoplegic migraine/ or exp migraine/ or exp migraine with aura/ or exp migraine without aura/ (57058)

10 ((withdrawal or overuse or "over use" or "over-use" or misuse or "mis-use" or abuse or induced) adj5 (medication* or medicine* or analges* or drug* or opiate* or opioid* or nsaids or non-opiate* or non opiate or ergot* or painkiller* or pain killer* or pain-killer*) adj5 (headache* or migraine*)).mp. (20637)

11 ((rebound or transformed) adj5 (headache* or migraine*)).ti,ab. (450)

12 8 or 9 or 10 or 11 (119498)

13 exp 'intermethod comparison'/ or exp 'data collection method'/ or exp 'validation study'/ or exp 'feasibility study'/ or exp 'pilot study'/ or exp 'psychometry'/ or exp 'reproducibility'/ or reproducib*.ab,ti. or 'audit'.ab,ti. or psychometr*.ab,ti. or clinimetr*.ab,ti. or clinometr*.ab,ti. or exp 'observer variation'/ or 'observer variation'.ab,ti. or exp 'discriminant analysis'/ or exp 'validity'/ or reliab*.ab,ti. or valid*.ab,ti. or 'coefficient'.ab,ti. or 'internal consistency'.ab,ti. or (cronbach* and ('alpha' or 'alphas')).ab,ti. or 'item correlation'.ab,ti. or 'item correlations'.ab,ti. or 'item selection'.ab,ti. or 'item selections'.ab,ti. or 'item reduction'.ab,ti. or 'item reductions'.ab,ti. or 'agreement'.ab,ti. or 'precision'.ab,ti. or 'imprecision'.ab,ti. or 'precise values'.ab,ti. or 'test-retest'.ab,ti. or ('test' and 'retest').ab,ti. or (reliab* and ('test' or 'retest')).ab,ti. or 'stability'.ab,ti. or 'interrater'.ab,ti. or 'inter-rater'.ab,ti. or 'intrarater'.ab,ti. or 'intra-rater'.ab,ti. or 'intertester'.ab,ti. or 'inter-tester'.ab,ti. or 'intratester'.ab,ti. or 'intra- tester'.ab,ti. or 'interobeserver'.ab,ti. or 'inter-observer'.ab,ti. or 'intraobserver'.ab,ti. or 'intra- observer'.ab,ti. or 'intertechnician'.ab,ti. or 'inter-technician'.ab,ti. or 'intratechnician'.ab,ti. or 'intra- technician'.ab,ti. or 'interexaminer'.ab,ti. or 'inter-examiner'.ab,ti. or 'intraexaminer'.ab,ti. or 'intra-examiner'.ab,ti. or 'interassay'.ab,ti. or 'inter-assay'.ab,ti. or 'intraassay'.ab,ti. or 'intra-assay'.ab,ti. or 'interindividual'.ab,ti. or 'inter-individual'.ab,ti. or 'intraindividual'.ab,ti. or 'intra-individual'.ab,ti. or 'interparticipant'.ab,ti. or 'inter-participant'.ab,ti. or 'intraparticipant'.ab,ti. or 'intra- participant'.ab,ti. or 'kappa'.ab,ti. or 'kappas'.ab,ti. or 'coefficient of variation'.ab,ti. or repeatab*.ab,ti. or ((replicab* or 'repeated') and ('measure' or 'measures' or 'findings' or 'result' or 'results' or 'test' or 'tests')).ab,ti. or generaliza*.ab,ti. or generalisa*.ab,ti. or 'concordance'.ab,ti. or ('intraclass' and correlation*).ab,ti. or 'discriminative'.ab,ti. or 'known group'.ab,ti. or 'factor analysis'.ab,ti. or 'factor analyses'.ab,ti. or 'factor structure'.ab,ti. or 'factor structures'.ab,ti. or 'dimensionality'.ab,ti. or subscale*.ab,ti. or 'multitrait scaling analysis'.ab,ti. or 'multitrait scaling analyses'.ab,ti. or 'item discriminant'.ab,ti. or 'interscale correlation'.ab,ti. or 'interscale

correlations'.ab,ti. or (('error' or 'errors') and (measure* or correlat* or evaluat* or 'accuracy' or 'accurate' or 'precision' or 'mean')).ab,ti. or 'individual variability'.ab,ti. or 'interval variability'.ab,ti. or 'rate variability'.ab,ti. or 'variability analysis'.ab,ti. or ('uncertainty' and ('measurement' or 'measuring')).ab,ti. or 'standard error of measurement'.ab,ti. or sensitiv*.ab,ti. or responsive*.ab,ti. or ('limit' and 'detection').ab,ti. or 'minimal detectable concentration'.ab,ti. or interpretab*.ab,ti. or (small* and ('real' or 'detectable') and ('change' or 'difference')).ab,ti. or 'meaningful change'.ab,ti. or 'minimal important change'.ab,ti. or 'minimal important difference'.ab,ti. or 'minimally important change'.ab,ti. or 'minimally important difference'.ab,ti. or 'minimal detectable change'.ab,ti. or 'minimal detectable difference'.ab,ti. or 'minimally detectable change'.ab,ti. or 'minimally detectable difference'.ab,ti. or 'minimal real change'.ab,ti. or 'minimal real difference'.ab,ti. or 'minimally real change'.ab,ti. or 'minimally real difference'.ab,ti. or 'ceiling effect'.ab,ti. or 'floor effect'.ab,ti. or 'item response model'.ab,ti. or 'irt'.ab,ti. or 'rasch'.ab,ti. or 'differential item functioning'.ab,ti. or 'dif'.ab,ti. or 'computer adaptive testing'.ab,ti. or 'item bank'.ab,ti. or 'cross-cultural equivalence'.ab,ti. (5156030)

14 (burden of migraine questionnaire or BURMIG or (beck depression inventory or BDI) or (comprehensive headache related quality of life questionnaire or CHQQ) or (completeness of response survey or CORS) or (cognitive impairment scale for migraine attacks or MIG-SCOG) or (chronic pain coping inventory or CPCI) or (depression anxiety stress scale or DASS) or (functional assessment in migraine or FAIM or FAIMQ) or (female sexual function index or FSFI) or (health utilities index or HUI) or (headache impact test or HIT 6 or HIT6 or HIT-6) or (headache impact score or HIS) or (headache management self-efficacy scale or HSES) or (headache-specific locus of control or headache specific locus of control) or (headache disability inventory or HDI) or (headache disability scale or HDS) or ((hospital anxiety and depression scale) or HADS) or ((headache-attributed restriction, disability, social handicap and impaired participation questionnaire) or HARDSHIP or HARDSHIPQ) or (headache activities of daily living index or HADLI) or (headache under response to treatment questionnaire or HURT or HURTQ) or (headache impact questionnaire or HIQ or HImQ) or (headache needs assessment survey or HANA) or (headache intensity or headache duration or headache severity) or (henry ford hospital headache disability inventory or HDI) or (impact of migraine-tension type headache-neck pain or Impact M-TTH-NP) or (italian perceived disability scale or IPDS) or (migraine treatment optimisation questionnaire or M-TOQ-15) or (migraine treatment satisfaction measure or MTSM) or (migraine-specific quality of life scale or MSQoL) or ((migraine work and productivity loss questionnaire) or MWPLQ) or (migraine disability assessment score or MIDAS) or (migraine disability assessment questionnaire or MDAS) or (migraine screen questionnaire or MS-Q) or (migraine impact questionnaire or MIQ) or (migraine specific quality of life questionnaire or MSQL or MSQ or MSQV 2 1) or (24-h MSQoLQ or 24-hour migraine specific quality of life questionnaire) or (patient perception of migraine questionnaire or PPMQ) or (patient health questionnaire or PHQ-9) or (pain disability index or PDI) or (pittsburg sleep quality index or PSQI) or (subjects global impression of change or SGIC) or (pain catastrophizing scale or PCS) or (visual aura rating scale or VARS) or (waters headache questionnaire or WHQ) or (numerical rating scale or NRS or numerical pain intensity scale or numerical pain rating scale or numeric rating scale for pain or NRS pain or NRS-pain) or (visual analogue scale or visual analogue scale for pain or VAS pain or VAS-pain or VAS) or (rating scale or analogue scale) or (SF36 or SF 36 or SF-36 or short form 36 or shortform 36 or short-form 36) or (SF12 or SF 12 or SF-12 or short form 12 or shortform 12 or short-form 12) or (SF6D or SF 6D or SF-6D or short form 6D or shortform 6D or short-form 6D) or (euroqol or euro qol or euro-qol or EQ5D or EQ 5D or EQ-5D)).tw. (552600)

15 (addresses or biography or case reports or comment or directory or editorial or festschrift or interview or lectures or legal cases or legislation or letter or news or newspaper article or patient education handout or popular works or congresses or consensus development conference or consensus development conference, nih or practice guideline).pt. not (*animals/ not *humans/) (1526497)

16 (12 and 13 and 14) not 15 (3250)

17 limit 16 to (human and english language and (adult <18 to 64 years> or aged <65+ years>)) (1794)

18 limit 17 to yr="1980 - 2016" (1760)

Database: Ovid MEDLINE(R) <1946 to April Week 2 2017>

Search Strategy:

----

1 Headache/ (26018)

2 exp headache disorders/ or exp headache disorders, primary/ (32133)

3 exp Tension-Type Headache/ (1860)

4 (headache* adj3 (mixed or combination or tension or tension type or muscle contraction* or psychomyogenic or stress or ordinary or essential or psychogenic)).tw. (4133)

5 ((chronic adj2 daily adj2 headache*) or (daily adj2 persistent adj2 headache*)).ti,ab. (950)

6 (headache* or hemicrania simplex).mp. (74687)

7 long term headache*.mp. (19)

8 chronic headache*.mp. (1518)

9 exp Headache Disorders/ (32133)

10 tension headache*.mp. (1004)

11 1 or 2 or 3 or 4 or 5 or 6 or 7 or 8 or 9 or 10 (88376)

12 (daily or persistent or chronic).mp. (1583660)

13 11 and 12 (17326)

14 migraine*.mp. or exp Migraine with Aura/ or exp Migraine Disorders/ or exp Ophthalmoplegic Migraine/ or exp Migraine without Aura/ (32683)

15 (withdrawal or overuse or "over use" or "over-use" or misuse or "mis-use" or abuse or induced).mp. adj5 (medication* or medicine* or analges* or drug* or opiate* or opioid* or NSAIDS or non-opiate* or non opiate* or ergot* or painkiller* or pain killer* or pain-killer*).ti,ab. adj5 (headache* or migraine*).ti,ab. (3474)

16 ((rebound or transformed) adj5 (headache* or migraine*)).ti,ab. (327)

17 13 or 14 or 15 or 16 (45932)

18 (instrumentation or methods).sh. or Validation Stud- ies.pt. or Comparative Study.pt. or exp Psychometrics/ or psychometr*.ti,ab. or clinimetr*.tw. or clino- metr*.tw. or exp "Outcome Assessment (Health Care)"/ or outcome assessment.ti,ab. or outcome measure*.tw. or exp observer variation/ or observer variation.ti,ab. or exp Health Status Indicators/ or exp reproducibility of results/ or reproducib*.ti,ab. or exp discriminant analysis/ or reliab*.ti,ab. or unreliab*.ti,ab. or valid*.ti,ab. or coefficient.ti,ab. or homogeneity.ti,ab. or homogeneous.ti,ab. or internal consistency.ti,ab. or (cronbach* and (alpha or alphas)).ti,ab. or (item and (correlation* or selection* or reduction*)).ti,ab. or agreement.ti,ab. or precision.ti,ab. or imprecision.ti,ab. or "precise values".ti,ab. or test- retest.ti,ab. or (test and retest).ti,ab. or (reliab* and (test or retest)).ti,ab. or stability.ti,ab. or interrater.ti,ab. or inter-rater.ti,ab. or intrarater.ti,ab. or intra-rater.ti,ab. or intertester.ti,ab. or inter-tester.ti,ab. or intratester.ti,ab. or intra-tester.ti,ab. or interobserver.ti,ab. or inter-observer.ti,ab. or intraobserver.ti,ab. or intra- observer.ti,ab. or intertechnician.ti,ab. or

inter-techni- cian.ti,ab. or intratechnician.ti,ab. or intra-technician.ti,ab. or interexaminer.ti,ab. or inter-examiner.ti,ab. or intraex- aminer.ti,ab. or intra-examiner.ti,ab. or interassay.ti,ab. or inter-assay.ti,ab. or intraassay.ti,ab. or intra-assay.ti,ab. or interindividual.ti,ab. or inter-individual.ti,ab. or intraindi- vidual.ti,ab. or intra-individual.ti,ab. or interparticipant.ti,ab. or inter-participant.ti,ab. or intraparticipant.ti,ab. or intra-participant.ti,ab. or kappa.ti,ab. or kappa-s.ti,ab. or kappas.ti,ab. or repeatab*.ti,ab. or ((replicab* or repeated) and (measure or measures or findings or result or results or test or tests)).ti,ab. or generaliza*.ti,ab. or general- isa*.ti,ab. or concordance.ti,ab. or (intraclass and correlation*).ti,ab. or discriminative.ti,ab. or "known group".ti,ab. or factor analysis.ti,ab. or factor analyses.ti,ab. or dimension*.ti,ab. or subscale*.ti,ab. or (multitrait and scaling and (analysis or analyses)).ti,ab. or item discriminant.ti,ab. or interscale correlation*.ti,ab. or error.ti,ab. or errors.ti,ab. or "individual variabil- ity".ti,ab. or (variability and (analysis or val- ues)).ti,ab. or (uncertainty and (measurement or measuring)).ti,ab. or "standard error of measure-ment".ti,ab. or sensitiv*.ti,ab. or responsive*.ti,ab. or ((minimal or minimally or clinical or clinically) and (important or significant or detectable) and (change or difference)).ti,ab. or (small* and (real or detectable) and (change or difference)).ti,ab. or meaningful change.ti,ab. or "ceiling effect".ti,ab. or "floor effect".ti,ab. or "Item response model".ti,ab. or IRT.ti,ab. or Rasch.ti,ab. or "Differential item functioning".ti,ab. or DIF.ti,ab. or "computer adaptive testing".ti,ab. or "item bank".ti,ab. or "cross-cultural equivalence".ti,ab. (5772794)

19 (burden of migraine questionnaire or BURMIG or (beck depression inventory or BDI) or (comprehensive headache related quality of life questionnaire or CHQQ) or (completeness of response survey or CORS) or (cognitive impairment scale for migraine attacks or MIG-SCOG) or (chronic pain coping inventory or CPCI) or (depression anxiety stress scale or DASS) or (functional assessment in migraine or FAIM or FAIMQ) or (female sexual function index or FSFI) or (health utilities index or HUI) or (headache impact test or HIT 6 or HIT6 or HIT-6) or (headache impact score or HIS) or (headache management self-efficacy scale or HSES) or (headache-specific locus of control or headache specific locus of control) or (headache disability inventory or HDI) or (headache disability scale or HDS) or ((hospital anxiety and depression scale) or HADS) or ((headache-attributed restriction, disability, social handicap and impaired participation questionnaire) or HARDSHIP or HARDSHIPQ) or (headache activities of daily living index or HADLI) or (headache under response to treatment questionnaire or HURT or HURTQ) or (headache impact questionnaire or HIQ or HImQ) or (headache needs assessment survey or HANA) or (headache intensity or headache duration or headache severity) or (henry ford hospital headache disability inventory or HDI) or (impact of migraine-tension type headache-neck pain or Impact M-TTH-NP) or (italian perceived disability scale or IPDS) or (migraine treatment optimisation questionnaire or M-TOQ-15) or (migraine treatment satisfaction measure or MTSM) or (migraine-specific quality of life scale or MSQoL) or ((migraine work and productivity loss questionnaire) or MWPLQ) or (migraine disability assessment score or MIDAS) or (migraine disability assessment questionnaire or MDAS) or (migraine screen questionnaire or MS-Q) or (migraine impact questionnaire or MIQ) or (migraine specific quality of life questionnaire or MSQL or MSQ or MSQV 2 1) or (24-h MSQoLQ or 24-hour migraine specific quality of life questionnaire) or (patient perception of migraine questionnaire or PPMQ) or (patient health questionnaire or PHQ-9) or (pain disability index or PDI) or (pittsburg sleep quality index or PSQI) or (subjects global impression of change or SGIC) or (pain catastrophizing scale or PCS) or (visual aura rating scale or VARS) or (waters headache questionnaire or WHQ) or (numerical rating scale or NRS or numerical pain intensity scale or numerical pain rating scale or numeric rating scale for pain or NRS pain or NRS-pain) or (visual analogue scale or visual analogue scale for pain or VAS pain or VAS-pain or VAS) or (rating scale or analogue scale) or (SF36 or SF 36 or SF-36 or short form 36 or shortform 36 or short-form 36) or (SF12 or SF 12 or SF-12 or short form 12 or shortform 12 or short-form 12) or (SF6D or SF 6D or SF-6D or short form 6D or shortform 6D or short-form 6D) or (euroqol or euro qol or euro-qol or EQ5D or EQ 5D or EQ-5D)).tw. (315280)

20 (addresses or biography or case reports or comment or directory or editorial or festschrift or interview or lectures or legal cases or legislation or letter or news or newspaper article or patient education handout or popular works or congresses or consensus development conference or consensus development conference, nih or practice guideline).pt. not (*animals/ not *humans/) (3636046)

21 (17 and 18 and 19) not 20 (1566)

22 limit 21 to (english language and humans and "all adult (19 plus years)") (1255)

23 limit 22 to yr="1980 - 2016" (1243)

Appendix 1.3 List of measures included in 'named PROM' searches (EMBASE and MEDLINE) (Total n= 51)

(burden of migraine questionnaire or BURMIG or

(beck depression inventory or BDI) or

(comprehensive headache related quality of life questionnaire or CHQQ) or

(completeness of response survey or CORS) or

(cognitive impairment scale for migraine attacks or MIG-SCOG) or

(chronic pain coping inventory or CPCI) or

(depression anxiety stress scale or DASS) or

(functional assessment in migraine or FAIM or FAIMQ) or

(female sexual function index or FSFI) or

(health utilities index or HUI) or

(headache impact test or HIT 6 or HIT6 or HIT-6) or

(headache impact score or HIS) or

(headache management self-efficacy scale or HSES) or

(headache-specific locus of control or headache specific locus of control) or

(headache disability inventory or HDI) or

(headache disability scale or HDS) or

((hospital anxiety and depression scale) or HADS) or

((headache-attributed restriction, disability, social handicap and impaired participation questionnaire) or

HARDSHIP or HARDSHIPQ) or

(headache activities of daily living index or HADLI) or

(headache under response to treatment questionnaire or HURT or HURTQ) or

(headache impact questionnaire or HIQ or HImQ) or

(headache needs assessment survey or HANA) or

(headache intensity or headache duration or headache severity) or

(henry ford hospital headache disability inventory or HDI) or

(impact of migraine-tension type headache-neck pain or Impact M-TTH-NP) or

(italian perceived disability scale or IPDS) or

(migraine treatment optimisation questionnaire or M-TOQ-15) or

(migraine treatment satisfaction measure or MTSM) or

(migraine-specific quality of life scale or MSQoL) or

((migraine work and productivity loss questionnaire) or MWPLQ) or

(migraine disability assessment score or MIDAS) or

(migraine disability assessment questionnaire or MDAS) or

(migraine screen questionnaire or MS-Q) or

(migraine impact questionnaire or MIQ) or

(migraine specific quality of life questionnaire or MSQL or MSQ or MSQV 2 1) or

(24-h MSQoLQ or 24-hour migraine specific quality of life questionnaire) or

(patient perception of migraine questionnaire or PPMQ) or

(patient health questionnaire or PHQ-9) or

(pain disability index or PDI) or

(pittsburg sleep quality index or PSQI) or

(subjects global impression of change or SGIC) or

(pain catastrophizing scale or PCS) or

(visual aura rating scale or VARS) or

(waters headache questionnaire or WHQ) or

(numerical rating scale or NRS or numerical pain intensity scale or numerical pain rating scale or numeric rating scale for pain or NRS pain or NRS-pain) or

(visual analogue scale or visual analogue scale for pain or VAS pain or VAS-pain or VAS) or

(rating scale or analogue scale) or

(SF36 or SF 36 or SF-36 or short form 36 or shortform 36 or short-form 36) or

(SF12 or SF 12 or SF-12 or short form 12 or shortform 12 or short-form 12) or

(SF6D or SF 6D or SF-6D or short form 6D or shortform 6D or short-form 6D) or

(euroqol or euro qol or euro-qol or EQ5D or EQ 5D or EQ-5D)).tw. (552600)

Appendix 2. Table 1: Multi-item PROMs identified (n=39) from full-text articles assessed for eligibility (searched 1980-2016); n=23 PROMs included in final review ('in-use' 2000-2016).

**PROM**	**Developer / article in which identified**	**Include / exclude from full review**	**Justification**	**Evaluations included in review (n)**	
				1988-1999	***Post-2000***
Migraine-specific (10)		***5/10***			
BURMIG questionnaire	*Andree, C., M. Vaillant, C. Rott, Z. Katsarava and P. S. Sandor (2008). "Development of a self-reporting questionnaire, BURMIG, to evaluate the burden of migraine." Journal of Headache & Pain 9(5): 309-315.*	*No*	*Developed in Swiss population – evidence of translation into English not clear in article. Initial evaluation in Swiss population. Exclude.*	-	*(1)*
Functional Assessment in Migraine Questionnaire (FAIM)	Pathak, D. S., D. J. Chisolm and K. A. Weis (2005). "Functional Assessment in Migraine (FAIM) questionnaire: development of an instrument based upon the WHO's International Classification of Functioning, Disability, and Health." Value in Health **8**(5): 591-600.	Yes	Fulfils inclusion criteria	-	1
Headache Needs Assessment (HANA) survey	Cramer, J. A., S. D. Silberstein and P. Winner (2001). "Development and validation of the headache needs assessment (HANA) survey." Headache **41**(4): 402-409.	Yes	Fulfils inclusion criteria	-	1
Migraine Disability Assessment Scale (MIDAS)	Stewart, W. F., R. Lipton, K. Kolodner, J. Liberman and J. Sawyer (1999A). "Reliability of the migraine disability assessment score in a population- based sample of headache sufferers." Cephalalgia **19**(2): 107-114.	Yes	Fulfils inclusion criteria	2	10
Migraine Quality of life Questionnaire (MQoLQ)	*Hartmaier, S. L., N. C. Santanello, R. S. Epstein and S. D. Silberstein (1995). "Development of a brief 24-hour migraine-specific quality of life questionnaire." Headache 35(6): 320-329.*	*No*	*No evaluations identified post-2000. Used as a comparator in establishing evidence in support of a new measure – the MTSM – but this is limited [32].*	*(1)*	*0*

Migraine-specific Quality of Life Questionnaire (MSQ) v1.	*Jhingran, P., J. T. Osterhaus, D. W. Miller, J. T. Lee and L. Kirchdoerfer (1998). "Development and validation of the migraine-specific quality of life questionnaire." Headache* ***38****(4): 295-302.*	*No*	*Succeeded by MSQ v2.1*	*(1)*	-
Migraine-specific Quality of Life Questionnaire (MSQ) v2.	*Jhingran, P., S. M. Davis, L. M. LaVange, D. W. Miller and R. W. Helms (1998). "MSQ: Migraine-specific quality-of-life questionnaire: Further investigation of the factor structure." PharmacoEconomics* ***13****(6): 707-717.*	*No*	*Succeeded by MSQ v2.1*	*(1)*	-
Migraine-specific Quality of Life Questionnaire (MSQ) v2.1.	Martin, B. C., D. S. Pathak, M. I. Sharfman, J. U. Adelman, F. Taylor, W. J. Kwong and P. Jhingran (2000). "Validity and reliability of the migraine-specific quality of life questionnaire (MSQ Version 2.1)." Headache **40**(3): 204-215.	Yes	Fulfils inclusion criteria	-	9
Migraine-specific Quality of Life Questionnaire (MSQoL)	Wagner, T. H., D. l. Patrick, B. S. Galer and R. A. Berzon (1996). "- A new instrument to assess the long-term quality of life effects from migraine: development and psychometric testing of the MSQOL." Headache **36**(8): 484-492.	Yes	Fulfils inclusion criteria	2	1
Migraine Symptom Frequency Bother questionnaire	*Patrick, D. L., M. L. Martin, D. M. Bushnell and J. Pesa (2003). "Measuring satisfaction with migraine treatment: expectations, importance, outcomes, and global ratings." Clinical Therapeutics* ***25****(11): 2920-2935.*	*No*	*Ad hoc measure developed specifically for single study. Not evaluated or applied again.*	-	*1*
Headache-specific (8)		**6/8**			
Comprehensive Headache Related Quality of Life Questionnaire (CHQQ)	*Manhalter, N., G. Bozsik, A. Palasti, E. Csepany and C. Ertsey (2012). "The validation of a new comprehensive headache-specific quality of life questionnaire." Cephalalgia* ***32****(9): 668-682.*	*No*	*Non-Anglicised evaluations (n=2)*	-	*(2)*

EUROLIGHT questionnaire	Andree, C., Vallaint M., Barre, J.,Katsarava R. et al. (2009). "Development and Validation of the EUROLOGHT questionnaire to evaluate the burden of primary headache disorders in Europe." Cephalalgia 30 (9):1082-1100.	Yes	Fulfils inclusion criteria	-	1
Headache Activities of Daily Living Index (HADLI)	Vernon, H. and G. Lawson (2015). "Development of the headache activities of daily living index: Initial validity study." Journal of Manipulative and Physiological Therapeutics **38**(2): 102-111.	Yes	Fulfils inclusion criteria	-	1
Headache Attributed Restriction, Disability, Social Handicap and Impaired<br><br>*Participation (HARDSHIP) questionnaire*	*Steiner, T. J., G. Gururaj, C. Andree, Z. Katsarava, I. Ayzenberg, S. Y. Yu, M. Al Jumah, R. Tekle-Haimanot, G. L. Birbeck, A. Herekar, M. Linde, E. Mbewe, K. Manandhar, A. Risal, R. Jensen, L. P. Queiroz, A. I. Scher, S. J. Wang and L. J. Stovner (2014). "Diagnosis, prevalence estimation and burden measurement in population surveys of headache: presenting the HARDSHIP questionnaire." Journal of Headache and Pain* ***15****(1).*	*No*	*Interview-administration only – modular instrument: demographic, diagnostic, headache-attributed burden – symptoms, health-care utilisation, disability, productive time loss, impact on education, career and earnings, control, relationships and family, qol, well-being, co-morbidities.*	-	-
Headache Disability Impact Questionnaire (HDI)	Niere K, Quin A. Development of a headache-specific disability questionnaire for patients attending physiotherapy. *Man Ther.* 2009 Feb;14(1):45-51	Yes	Fulfils inclusion criteria	-	1
Headache Impact Test (HIT)	Bjorner, J. B., M. Kosinski and J. E. Ware Jr (2003A). "Calibration of an item pool for assessing the burden of headaches: An application of item response theory to the Headache Impact Test (HITTM)." Quality of Life Research **12**(8): 913-933.	Yes	Fulfils inclusion criteria	-	3
Headache Impact Test-6 (HIT-6)	Bjorner, J. B., M. Kosinski and J. E. Ware Jr (2003C). "Using item response theory to calibrate the Headache Impact Test (HITTM) to the metric of traditional headache scales." Quality of Life Research **12**(8): 981-1002.	Yes	Fulfils inclusion criteria	-	12
Henry Ford hospital headache disability inventory (HDI).	*Jacobson, G. P., N. M. Ramadan, S. K. Aggarwal and C. W. Newman (1994). "The Henry Ford hospital headache disability inventory (HDI)." Neurology* ***44****(5): 837-842.*	*No*	*No evaluations identified post-2000*		-

SF-36 ‘Headache-specific’ Modification	Magnusson JE, Riess CM, Becker WJ. Modification of the SF-36 for a headache population changes patient-reported health status. *Headache.* 2012; 52(6): 993-1004.	Yes	Fulfils inclusion criteria	-	1
Response to treatment (7)		**6/7**			
Completeness of Response Survey (CORS)	Coon, C. D., S. E. Fehnel, K. H. Davis, M. C. Runken, M. E. Beach and R. K. Cady (2012). "The development of a survey to measure completeness of response to migraine therapy." Headache **52**(4): 550-572.	Yes	Fulfils inclusion criteria	-	1
Migraine Assessment of Current Therapy (Migraine-ACT) Questionnaire	Dowson, A. J., S. J. Tepper, V. Baos, F. Baudet, D. D'Amico and S. Kilminster (2004). "Identifying patients who require a change in their current acute migraine treatment: The Migraine Assessment of Current Therapy (Migraine-ACT) questionnaire." Current Medical Research and Opinion **20**(7): 1125-1135.	Yes	Fulfils inclusion criteria	-	2
Migraine Therapy Assessment Questionnaire<br><br>(M-TAQ)	Chatterton ML[1], Lofland JH, Shechter A, Curtice WS, Hu XH, Lenow J, Smullens SN, Nash DB, Silberstein SD.<br><br>Reliability and validity of the migraine therapy assessment questionnaire. Headache. 2002 Nov-Dec;42(10):1006-15.	Yes	Fulfils inclusion criteria	-	1
Migraine Therapy Optimisation Questionnaire<br><br>(M-TOQ)	Lipton, R. B., K. Kolodner, M. E. Bigal, D. Valade, M. J. A. Lainez, J. Pascual, A. Gendolla, G. Bussone, N. Islam, K. Albert and B. Parsons (2009). "Validity and reliability of the migraine-treatment optimization questionnaire." Cephalalgia **29**(7): 751-759.	Yes	Fulfils inclusion criteria	-	1
Migraine Treatment Satisfaction Measure (MTSM)	Patrick, D. L., M. L. Martin, D. M. Bushnell and J. Pesa (2003). "Measuring satisfaction with migraine treatment: expectations, importance, outcomes, and global ratings." Clinical Therapeutics **25**(11): 2920-2935.	Yes	Fulfils inclusion criteria	-	2

Patient Perception of Migraine Questionnaire (PPMQ)	*Davis, K. H., L. Black and B. Sleath (2002). "Validation of the Patient Perception of Migraine Questionnaire." Value in Health **5**(5): 422-430.*	*(Yes)*	*Fulfils inclusion criteria – but succeeded by PPMQ-R*	-	*1*
Patient Perception of Migraine Questionnaire – Revised (PPMQ-R)	Revicki, D. A., M. Kimel, K. Beusterien, J. W. Kwong, J. A. Varner, (2006).	Yes	Fulfils inclusion criteria	-	2
**Generic (8)**		**6/8**			
Generic quality of life / health status					
Profile measures (4)		**3/4**			
Short Form Health Survey 8 (SF-8)	Turner-Bowker, D. M., M. S. Bayliss, J. E. Ware Jr and M. Kosinski (2003). "Usefulness of the SF-8TM Health Survey for comparing the impact of migraine and other conditions." Quality of Life Research **12**(8): 1003-1012.	Yes	Fulfils inclusion criteria	-	4
Short Form Health Survey 12 (SF-12)	Lipton, R. B., S. W. Hamelsky, K. B. Kolodner, T. J. Steiner and W. F. Stewart (2000). "Migraine, quality of life, and depression: a population-based case-control study." Neurology **55**(5): 629-635.	Yes	Fulfils inclusion criteria	-	1
Short Form Health Survey 36 (SF-36)	Solomon, G. D., F. G. Skobieranda and L. A. Gragg (1993). "Quality of life and well-being of headache patients: measurement by the medical outcomes study instrument." Headache **33**(7): 351-358.	Yes	Fulfils inclusion criteria	-	5
World Health Organisation Disability Assessment II (WHO-DAS II)	*Raggi, A., M. Leonardi, G. Bussone and D. D'Amico (2011). "Value and utility of disease-specific and generic instruments for assessing disability in patients with migraine, and their relationships with health-related quality of life." Neurological Sciences 32(3): 387-392.*	*No*	*Non-Anglicised evaluations (n=3)*	-	*3*

Utility measures (4)		**3/4**			
EuroQol EQ-5D-3L	Essink-Bot, M. L., P. F. Krabbe, G. J. Bonsel and N. K. Aaronson (1997). “An empirical comparison of four generic health status measures. The Nottingham Health Profile, the Medical Outcomes Study 36-item Short-Form Health Survey, the COOP/WONCA charts, and the EuroQol instrument.” Medical care **35**(5): 522-537. *Non-Anglicised evaluation*<br><br>*Anglicised evaluations included in review (n=3)*	Yes	Fulfils inclusion criteria	-	3
Health Utilities Index (HUI)	*Mo, F., B. C. Choi, F. C. Li and J. Merrick (2004). "Using Health Utility Index (HUI) for measuring the impact on health-related quality of Life (HRQL) among individuals with chronic diseases." The Scientific World Journal **4**: 746-757.*	*No*	*Succeeded by HUI-3*	-	*1*
Health Utilities Index-3 (HUI-3)	Brown, J. S., P. J. Neumann, G. Papadopoulos, G. Ruoff, M. Diamond and J. Menzin (2008). "Migraine frequency and health utilities: findings from a multisite survey." Value in Health 11(2): 315-321.	Yes	Fulfils inclusion criteria	-	(1)
Quality of Well Being Scale (QWB-8)	Sieber, W. J., K. M. David, J. E. Adams, R. M. Kaplan and T. G. Ganiats (2000). "Assessing the impact of migraine on health-related quality of life: An additional use of the quality of well-being scale-self-administered." Headache **40**(8): 662-671.	Yes	Fulfils inclusion criteria	-	1

Appendix 3: Table 2: Assessment criteria for the quality of reported measurement properties [17,19,20].

Measurement property	Rating	Assessment of quality
**Reliability**		
Internal consistency	+	Cronbach's alpha(s) > 0.70
- *the extent to which items within a measure are*	?	Cronbach's alpha not evaluated or dimensionality unknown
internally consistent	-	Cronbach's alpha(s) < 0.70
**Reliability**	+	Intra-class Correlation Coefficient (ICC)/weighted Kappa >0.70 OR Pearson's r >0.80
(test-retest / inter-rater / inter-rater)	?	Neither ICC/weighted Kappa, not Pearson's r evaluated
- *the extent to which a measure provides the same results on repeated completions, assuming no change in the underlying health state*	-	ICC/weighted Kappa <0.70 OR Pearson's r <0.80
**Validity**		
Content validity - *the extent to which the item content of a measure is an adequate reflection of the construct being measured*	+	Authors provide a clear description of the measurement aim, target population, concept(s) measured and process of item selection. Members of the target population and experts in the field were clearly identified as being involved in development. For measures applied for the first time in a new population, evidence that the views of members of the target population (and experts in the field) have been sought to determine relevance, comprehension and comprehensiveness.
	?	Insufficient evidence available
	-	No detail re measurement aim, target population, concept(s) measured, process of item selection; members of the target population or experts were not specifically involved in development.<br>For measures applied for the first time in a new population, evidence whereby the relevance and acceptability of the measure with members of the target audience or experts was not provided.
Construct validity - Structural validity	+	Factors should explain 50% of the variance
- *the extent to which PROM scores adequately reflect the*	?	Explained variance not reported
dimensionality of the construct being measured.	-	Factors explain < 50% of the variance
Construct validity - Hypothesis testing - *convergent (the extent to which measures of related*	+	Correlations with measures of the same construct should be >0.50 OR at least 75% of the results in accordance with hypothesized associations AND correlations with related constructs should be higher than with those reported with unrelated constructs
constructs are related to each other)	?	Only report correlations with unrelated constructs

- *discriminant (the extent to which a measure can demonstrate differences between groups known to differ on important variables)*	-	Correlations with measures of the same construct are <0.50 OR < 75% of the results in accordance with hypothesized associations OR correlations with related constructs are lower than those reported with unrelated constructs
**Responsiveness**<br>- *the ability to detect important change over time in the construct being measured (criterion / construct-based assessment)*	+	Change-score correlations with measures of the same construct are >0.50 OR at least 75% of the results are in accordance with hypothesized associations OR the Area Under the Curve (AUC) is >0.70 AND change-score correlations with measures of related constructs are higher than those reported with unrelated constructs
	?	Solely correlations with unrelated constructs
	-	Change-score correlations with measure of the same construct <0.50 OR < 75% of the results are in accordance with hypothesized associations OR AUC is <0.70 AND change-score correlations with related constructs are lower than those reported with unrelated constructs

Appendix 4: Table 3 Characteristics of reviewed PROMs evaluated in the headache population (total = 23)

**PROM**<br>***(Author; web-link[b]; completion format)[c]***	**Items n[a]**	**Construct**<br><br>**Domains (items)**	**Response options (range)**	**Recall Period**	**Score range**	**Administration**
Condition-specific (17)						*Time*
Migraine-impact (5/17)						
Functional Assessment in Migraine (FAIM)<br><br>(Pathak et al, 2005)[33]<br><br>*Self-completion*<br>*Items listed in development paper*	9 + 5	Underpinned by the WHO ICF. Focus on the functional impact of migraine.<br><br>3 domains: Two mental function:<br>Attention/Thought (5): concentration, control of life, focus on issues, spontaneity, think quickly.<br>Perception (4): find a peaceful place, light/sound/interaction avoidance.<br>One overall domain: 'Activity and Participation'(5): select up to 5 items from list of 28.	Item stem: How much does each item impact on their lives?<br><br>7-point scale: 1= 'not at all' to 7 – 'all of the time'	Within 24-hours of their typical migraine onset	Item summation within the three domains.<br><br>A/T: range 5-35<br>P: range 4-28<br>A/P: range 5-35<br><br>Lower scores indicate less functional impact.	5-10 minutes<br><br>*Not reported in headache population*
Headache Needs Assessment (HANA)<br><br>(Cramer et al,2001)[34]<br><br>*Self-completion*<br>*Copy of PROM in appendix to paper*	7	Migraine quality of life – frequency and bothersomeness (7):<br>Anxiety/worry; depression/discouragement; self-control; energy; function/work; family/social activities; overall impact of migraines.	For each item:<br>Frequency: How often has this problem occurred?: never / rarely / sometimes / often / all the time)<br>Bothersomeness: How much has this problem bothered you?: not at all / a little / some / a lot / a great deal.	Not stated	Item summation.<br>Total range 7-175, where lower scores indicate less impact.	5-10 minutes<br><br>*Not reported in headache population*
Migraine Disability Assessment Score (MIDAS)<br><br>(Stewart et al. 1999)[35]<br><br>http://www.achenet.org/midas/	12	Migraine disability (*but attribution is 'headache')*<br><br>3 domains (5 scored items)<br>Missed days/ reduced productivity at paid work (2)	Item stem: About ALL of the headaches you have had…<br><br>Frequency - number of days/ half days of disability	3 months	Total score derived as sum of lost days, where greater number of days indicates greater migraine-related disability.	5-10 minutes<br><br>*Not reported in headache population*

Self-completion		Missed days/ reduced productivity at household work (2)<br>Missed non-work activities (1)<br><br>Plus: 2 unscored items<br>Frequency of headaches *(how many days?)* (1)<br>Headache pain severity (1)	Not scored:<br>Number of days<br><br>Scale 0-10 (0= no pain at all, and 10= pain as bad as can be)			
Migraine-Specific Quality of Life Questionnaire version 2.1<br><br>(MSQ v2.1)<br><br>(Martin et al, 2000 [36]<br><br>http://www.outcomes-trust.org/instruments.htm#msql2.1<br><br>*Contact for further details and copy of questionnaire:* michael.c.runken@gsk.com<br><br>*Self-completion*	9	Health-related quality of life - impairments attributed to migraine<br><br>3 domains (14 items)<br>Role function – Restrictive (RR)(7): social – family/friends; leisure (2); work/ADL(3); cognition (1); symptoms: fatigue (1)<br><br>Role function – Preventive (RP)(4): symptoms: fatigue (1); work /ADL (2); social (1)<br><br>Emotional function (EF)(3): frustration (1); feeling a burden (1); letting others down (1)	Item stem: How often have migraines interfered with / limit/ed your ability to / keep you from getting as much done / had difficulty in /<br><br>How often have you had to cancel / need help / have to stop / not able to go… … because of your migraine<br><br>6-point categorical scale:<br>None of the time (1), A little bit of the time (2), Some of the time (3), A good bit of the time (4), Most of the time (5), All of the time (6).	4 weeks	Items summed within the three domains and transformed to 0-100 scale.<br><br>Higher scores indicate a worse quality of life	5-10 minutes<br><br>*Not reported in headache population*
Migraine-Specific Quality of life (MSQOL) measure<br><br>(McKenna et al, 1998) [37]	1	Needs-based migraine-specific quality of life<br><br>3 domains (20 items):	Item stems: Various – include: 'I try to avoid …' / 'It's important for	Responders advised to 'choose the answer that	All items summed (score range 20-80) and transformed to 0-100 scale.	5-10 minutes

http://www.galen-research.com/content/measures/MSQoL%20UK%20-%20First%20page%20sample.pdf<br><br>*Payment required to access full version of the questionnaire*<br><br>*Self-completion*		Avoidance behaviours (10)<br><br>Social relations (6)<br><br>Feelings (4)	me…' / 'I feel helpless …'/ 'I worry about …'<br><br>4-point categorical response scale: range:<br>Yes, very much / I try very hard / very important (1)<br>Yes, quite a lot / I try quite hard / quite important (2)<br>A little / I do not try very hard / not very important (3)<br>Not at all / I do not try at all / it's not important at all (4)	applies to you: between migraine attacks OR with any treatment you use now.	Higher scores indicate better quality of life	*Not reported in headache population*
Headache-impact (6/17)						
EUROLIGHT<br><br>(Andree et al, 2010)[28]<br><br>http://www.l-t-b.org/index.cfm/spKey/publications.html<br><br>*Self-completion*	1	Burden of primary headache disorders.<br><br>Includes assessment of headache characteristics, co morbidities, disease management and quality of life<br><br>6 sections (103 items):<br>1) Biographical (age, gender, language and employment)<br>2) Screening questions for headache (life-time and 1-year prevalence)<br>3) Diagnostic questions - based on the criteria of the *International Classification of Headache Disorders (*ICHD-II)<br>4) Questions about any headache experienced 'yesterday' (point prevalence)<br>5) Use of healthcare resources (medicines, investigations, consultations, etc.)	All domains categorical response categories – various number of options	Headache frequency in past month/yesterday<br><br>Healthcare past 30 days<br><br>Headache impact on 'own life'<br><br>Headache-related lost time in past 3 months	Various<br><br>As per WHOQoL, HALT-index and HADS.	Not reported.<br><br>Will require considerable completion time

	1	6) Impact of headache on work, family life and social activities: includes items taken from the WHOQOL (8-items), the HALT-index and HADS.				
Headache Activities of Daily Living Index<br><br>(HADLI)<br><br>(Vernon & Lawson, 2015) [29]<br><br><br>*Self-completion*	1	Headache-related 'activity disability' - ability of an individual to engage with usual activities of daily life during headache episode<br><br>1 domain (9 items):<br>Personal care<br>Lifting<br>Reading (including computers)<br>Sleeping (over last week)<br>Exercising (over last week)<br>Social activities<br>Work<br>Driving or travelling<br>Recreation	6-point categorical response options, where 0 is best ability and 5 is worst ability.	During headache episode 'when you have a headache'	Item summation: score range 0 to 45, where 45 is maximum activity disability.<br><br>Total score converted to percentage	3 minutes
Headache Disability Questionnaire<br><br>(HDQ)<br><br>(Niere & Quin, 2009) [30]<br><br><br>*Self-completion*	1	Headache specific disability in patients presenting for physiotherapy treatment<br><br>3 domains (9 items):<br>Pain (2): usual pain intensity; when pain is severe.<br><br>Activity Limitation (4):<br>Decreased efficiency in non-work activities<br>Decreased ability to work/study<br>Decreased efficiency in housework or chores<br>Proportion of times when work is missed<br><br>Activity Prevention (3):<br>Number of days where chores prevented	11 point Numerical Rating Scales<br><br>Anchors (0-10):<br>Adjectival anchors:<br>No pain (0) - Worst pain (10)<br>Never (0) – Always (10)<br>None (0) – Everyday (10)<br>Not reduced (0) - Unable to work (10)<br>Not reduced (0) - Unable to perform (10)	Past 1-month	Item summation. Index range 0-90, where higher scores indicate greater headache-specific disability.	??

		Number of days non-work activities prevented<br>Number of days in last month when had to lie down for >1 hour;				
Headache Impact Test (HIT)<br><br>CAT-HIT<br>*(IRT-HIT)*<br><br>(Bjorner JB et al. 2003a [3]; Ware JE et al, 2003 [57])<br><br>*Self-completion- requires internet interface for CAT completion*	3	Headache impact<br><br>Items, derived from four established measures:<br>MIDAS<br>MSQ (v1.0)<br>Headache Disability Index (HDI)<br>Headache Impact Questionnaire (HIMQ)<br><br>Plus experimental items generated from clinical trial data, and consultation with clinicians.<br><br>One domain 'Headache Impact': 54-item 'item bank'<br>Items cover a wide spectrum of headache impact, including minor headache.<br><br>Items cover of pain, role and social functioning, energy/fatigue, cognitive function, and mental health	Up to five categorical responses<br><br>Internet completion only using Computerized Adaptive Testing (CAT): CAT-HIT	1-month	Scored using Item Response Methods (IRT) *(also referred to as IRT-HIT).*<br><br>Number of completed items determined by 'stopping rule': mean number 6/54 items.<br><br>Norm-based scoring with mean 50 (SD 10), where higher scores indicate very severe headache impact.	Approximate response times of 1.5 minutes for those with least headache impact (HIT scores < 50)<br><br>Range from 2.4 items/ minute for a 9-item survey to 3.3 items/minute for a 6-item survey
Headache Impact Test<br>(HIT-6)<br><br>(Kosinski et al, 2003a) [31]<br><br>https://www.optum.com/optum-outcomes/what-we-do/disease-specific-health-surveys/hit-6.html<br><br>*Self-completion*	8	Headache impact<br><br>Static, short-form HIT: 6 domains (6-items)<br>Pain (*headache - how often is the pain severe*?) (1)<br>Social functioning (*limit your ability to do usual daily activities – work / adl / social?)* (1)	Equally weighted 5-option categorical scale with specific item score (generated to closely match the IRT score)<br><br>Item stem: *'how often…?'*<br><br>Attribution: *'when you have a headache /*	3 items (Vitality; Psychological distress; Cognition): past 4weeks<br><br>2 items (Pain. Role limitation): 'when you have a headache'	Item summation to create index score: range 36-78<br><br>Score interpretation (norm-based mean 50 (SD 10)):<br>>60: very severe impact<br><br>56-59: substantial impact	

		Role functioning *(how often do you wish you could lie down?)* (1)<br>Vitality *(too tired to do work or adl?)* (1)<br>Cognitive functioning *(limit ability to concentrate on work or adl?)* (1)<br>Psychological distress *(felt fed up or irritated)* (1)	*because of your headache'*<br><br>Range *(weighted item responses):*<br>Never (6 points), Rarely (8 points), Sometimes (10 points), Very Often (11 points), Always (13 points)	1 item (Social function): how often do headaches limit …	50-55: some impact<br><br>49 or <: little or no impact	
'Headache' SF-36<br><br>(Magnusson J.E. et al. 2012)[32]<br><br>*Self-completion*	1	Headache-related health status<br><br>Modification of original SF-36 to 'improve applicability' to the headache population by inserting 'including your headaches' to 6/36 items: physical functioning (item 3); role limitation - physical (item 4); social functioning (items 6 and 10); bodily pain (items 7 and 8)	Equally weighted 3 or 5 point Likert Scale	4 weeks or 1 week	Scale scores transformed to 0-100 calibrated at 50 as the norm<br><br>8 domains<br>2 summary scales	5-10 minutes
Response to migraine-specific treatment (6/17)						
Completeness of Response to Migraine Therapy Survey<br><br>(CORS)<br><br>(Coon CD. et al. 2012) [38]<br><br>*Both versions of CORS illustrated in full in appendix to article*<br><br>*Self-completion*	1	Optimal treatment: considers factors important to patients when considering the initiation/continuation of migraine treatment<br><br>Two modules:<br><br>1.'Static CORS' – to evaluate treatment at a single time-point<br><br>5 domains (24 items):<br><br>Frequency of Complete Relief (FCR) (6 – *how often complete*	*Item stem and response options:*<br>1.'Static CORS': categorical response options range 3, 4 or 5-options:<br><br>FCR: *how often does your current M Rx*	'overall experience with your current migraine treatment'	1.'Static CORS': three domains scores based on item summation<br><br>FCR (6): 1a,2b,3b,4b,5b,6b<br>SCR (6): 1b,2c,3c,4c,5c,6c<br>SRF (4): 7,8,9,10<br><br>- where higher scores indicate better medication response<br><br>2.'Comparative CORS': item summation to	10-15 minutes<br><br>*Not reported in headache population*

		relief of symptoms* plus irritability /moodiness**)<br><br>Speed of Complete Relief (SCR) (6 - *how quickly complete relief of* symptoms* plus irritability/ moodiness**)<br><br>Speed of Return to Functionality (SRF)(4 – able to concentrate/ think; normal activities; functioning normally (100%); feeling completely normal (100%))<br><br>Frequency of Migraine Recurrence (FMR)(1)<br><br>Confidence in Treatment (CIT)(2)<br><br>5 additional items describe presence of symptoms*:<br><br>24-items address:<br>Symptoms* - Headache-specific pain; neck/shoulder pain; nausea; sensitivity to light; sensitivity to sound (5 items).<br>Emotional well-being – **irritability or moodiness<br>Cognition – ability to ‘concentrate or think’<br>ADL – resumption of normal activities<br>Function – resumption of normal functioning (100%)<br>Feeling ‘completely normal’ (100%)<br>Confidence in current medical: frequency of return of M	*completely relieve your X?:* 5-point (0-4): range 0= none of the time, to 4 = all or almost all of the time.<br><br>SCR: *how quickly does your current M Rx completely relieve your X?:* 4-point (1-4): range 4 = < 30 mins, to 1 = > 2hrs<br><br>SRF: *how quickly are you able to … after taking your current M RX?:* 5-point (1-5): range 5 = < 30 mins, to 1 = > 4hrs<br><br>FMR: 5-point (0-4): range 0= none of the time, to 4 = all or almost all of the time.<br>CIT: 3-point (0-2): range 0 = not at all confidence, 1 = somewhat confident; to 2 = very confident.		produce index score (range 8 to 40), where higher scores suggest a better response to current medication than previous	

		within 24hrs; confidence that Rx will completely relieve M; confidence that M will not come back.<br><br>2. 'Comparative CORS (8 items) – for the comparative evaluation of two treatments at a single time-point.<br><br>8 domains (8 items):<br>Completeness of Relief (1)<br>Speed of Relief (1)<br>Persistence of Relief (1) (e.g. prevented symptoms from coming back within 24-hrs)<br>Return to Normal Function (1)<br>Fatigue (1)<br>Confidence in Treatment (2): that one does would completely relieve M within 2-hrs; that M would not come back within 24-hrs<br><br>Overall Satisfaction (1) – most satisfied	2. 'Comparative CORS': *Which medication provided the complete / quicker / longer-lasting / allowed more normal function / experience less fatigue / feel more confidence that one does would completely relieve your M within 2 hours?:*<br>5-point categorical scale (1-5); where a score of '1' favours the previous medication; a score of 3 suggests no preference between medications; a score of 5 favours the study medication.			
Migraine – Assessment of Current Therapy<br><br>(M-ACT) *Copy included in publication*<br><br>(Dowson et al, 2004)[39]<br><br>*Self-completion*	2	4 domains (27 long-form/ 4 short):<br>Headache impact (11/1)<br>Global assessment of relief (9/1)<br>Consistency of response (3/1)<br>Emotional response (4/1)	Item stem: 'When you take your treatment:<br>Dichotomous answers: Yes/No, where Yes = 1 and No = 0	Varies: between 2hours and 48-hours	Item summation	5-10 minutes<br><br>*Not reported in headache population*
Migraine Therapy Assessment Questionnaire<br><br>(M-TAQ)	1	To identify barriers to optimal migraine management and improve patient outcomes	Dichotomous answers: Yes/No,	Varies – mostly 2 hours.	Item summation<br>Range 0 to 8 (items 3 and 4 scored together),	5-10 minutes

(Chatterton et al, 2002)[40]<br><br>*Self-completion*<br>*Copy of PROM included in appendix to paper*		A disease-management/screening tool to identify individuals whose migraine management is sub-optimal (9)<br>Migraine control, frequency of attacks, knowledge and behavioural barriers, economic burden, treatment satisfaction	where Yes = 1 and No = 0		where higher scores indicates greater number of migraine issues<br><br>Also 3 domains:<br>Migraine control;<br>Knowledge/behaviour /treatment satisfaction;<br>Economic burden,	*Not reported in headache population*
Migraine-Treatment Optimization Questionnaire<br><br>(M-TOQ)<br><br>(Lipton RB. et al. 2009) [41]<br><br>*Self-completion*	1	Aims to support treatment optimization – defined as the achievement of realistic treatment goals.<br><br>The M-TOQ was developed to provide a rapid assessment of migraine therapy for use in primary care settings<br><br>M-TOQ 15: 5 domains (15 items):<br>Functioning (3)<br>Rapid relief of headache (3)<br>Consistency of response (3)<br>Prevention of recurrence (3)<br>Side effects (3)<br><br>M-TOQ 5: 5 domains (5 items)<br>Functioning (1)<br>Rapid relief of headache (1)<br>Consistency of response (1)<br>Prevention of recurrence (1)<br>Side effects (1)	Dichotomous answers: Yes/No, where Yes = 1 and No = 0	Last 4 weeks	M-TOQ 15<br>Item summation producing five domain scores or an index score. High scores suggest good response to treatment, suggesting that treatment change is *unlikely* to be required.<br><br>M-TOQ 5<br>Item summation producing an index score. If answer 'Yes' to all five items - treatment is considered satisfactory.<br>If answer 'no' to any single question, a change in treatment should be considered.<br><br>*A 'treatment optimization' table is provided to support score interpretation and clinical decision-making (Table 5)[24]*	5-10 minutes<br><br>*Not reported in headache population*
Migraine Treatment Satisfaction Measure<br><br>(MTSM)	2	Migraine treatment satisfaction	TE-M: 5-point response scale (1-5) where 1 is the worst	At onset and 24 hours after migraine episode	Overall 'MTSM' treatment satisfaction score is generated as	15-20 minutes

(Patrick et al, 2003) [42]<br><br>*Self-completion*		4-part assessment:<br>1) Expectations of Treatment for Migraine (TE-M); 9 items) – worded to express the 9 attributes as an 'expectation'.<br>2) Importance Ranking for Migraine Treatment (IR-M); 9 items) – respondents rank items to express their 'desired expectations; for each attribute.<br>3) Outcomes of Treatment for Migraine (TO-M); 9-items – correspond to the TE-M items) – produces a self-report of treatment outcome for each attribute.<br>4) Satisfaction with Migraine Treatment (PST-M; 9-items) – reflect satisfaction with treatment outcome across the 9 attributes.<br><br>9 attributes ('items') associated with migraine relief:<br>Pain relief<br>Speed of relief<br>Freedom from pain<br>Additional symptoms<br>Confidence in treatment<br>Disruption in life<br>Dosing<br>Freedom from relapse<br>Ease of use	case scenario (eg, no relief) and 5 is the best (eg total relief).<br><br>IR-M: ranking items on a 10cm line (where 0 = not important and 10 = most important). Intersection with the line = score (range 0-10).<br><br>TO-M: 5-point response scale (1-5) reflecting actual outcome, where 1 =-worst case scenario (eg no relief) to 5 = best case (eg total relief).<br><br>PST=M: 10cm VAS where 0 = most dissatisfied and 10 = most satisfied.		the sum of the nine derived attribute scores (a detailed scoring procedure is detailed by the developers [27]): score represents patients expectations about Rx, modified by Rx experience, weighted by their adjusted importance values, and used to modify the raw satisfaction values [27].<br><br>Scores also calculable for three domains:<br>Expectations (TE-M)<br>Outcomes (TO-M)<br>Satisfaction (PST-M)	*Not reported in headache population*
Patient Perception of Migraine Questionnaire -Revised<br><br>(PPMQ-R)<br><br>(Revicki DA et al. 2006)[43]	2	Patient satisfaction with acute migraine therapy<br><br>Core: 4 domains (19 items)<br>Efficacy: satisfaction with treatment efficacy (11)<br>Function: ability to perform usual activities (4)<br>Ease of Use (2)	Core domains 1 to 4 and 3 global items: 7-point 'Likert' scale: range 1 = very satisfied to 7 = very dissatisfied.<br><br>Domain 5 – 'Bothersomeness': 5-	4 weeks	Item summation to create domain scores:<br>Domain 1: range 11 to 77<br>Domain 2: range 4 to 28<br>Domain 3: range 2 to 8<br>Domain 4: range 2 to 8: where lower scores	

		Cost (2) (*may be removed where 'cost' is not a consideration*).<br><br>Additional fifth domain (10 items):<br>'Bothersomeness' or 'Tolerability' - related to side effects (10)<br><br>3 global items:<br>Overall satisfaction with medication effectiveness<br>Side effects<br>General treatment	point 'Likert' scale: range 1 = not at all, to 5 = extremely.		suggest greater satisfaction with treatment.<br><br>Item summation of three core domains (Efficacy, Function and Ease) to produce 'Total Satisfaction Score': score transformed to 0-100, where higher scores represent greater satisfaction.	
Generic measures (6)						
Profile measures (3/6)						
Short Form 36-item Health Survey<br><br>(SF-36) (version 1 (v1))<br><br>[Ware et al, 1994] [44]<br><br>https://campaign.optum.com/content/optum/en/optum-outcomes/what-we-do/health-surveys.html<br><br>*Self-completion or interview administered*		General health status<br><br>8 domains (36 items)<br>Bodily pain (BP)(2)<br>General health (GH)(5)<br>Mental health (MH) (5)<br>Physical functioning (PF)(10)<br>Role limitation-emotional (RE)(3)<br>Role limitation-physical (RP)(4)<br>Social functioning (SF)(2)<br>Vitality (V)(4)	Categorical: 2-6 options	Recall:<br>Standard 4-weeks<br>Acute 1-week	Requires algorithm to score domains<br><br>Norm-based scoring: score transformed to 0-100 (mean 50 (SD 10))<br><br>Individual domain scores ('profile') or<br>2 summary scales:<br>Physical Component Summary<br>Mental Component Summary	15 to 30 mins<br><br>*Not reported in headache population*
Short Form 12-item Health Survey<br><br>(SF-12)(v1)<br><br>[Ware et al, 1995][45]<br><br>https://campaign.optum.com/content/optum/en/optum-outcomes/what-we-do/health-surveys.html	1	Health Status<br><br>8 domains (12 items)<br>Physical functioning ((n items per domains???)<br>Social functioning<br>Role physical<br>Bodily pain	Categorical: 2-6 options	Recall:<br>Standard 4-weeks<br>Acute 1-week	Requires algorithm to score domains<br><br>Norm-based scoring: score transformed to 0-100 (mean 50 (SD 10))<br><br>2 summary scales:	5-15 minutes<br><br>*Not reported in headache population*

Self-completion or interview administered		Mental health<br>Role emotional<br>Vitality<br>General health			Physical Component Summary<br>Mental Component Summary	
Short Form 8-item Health Survey<br><br>(SF-8)(v1)<br><br>[Ware et al, 2001][46]<br><br>https://campaign.optum.com/optum-outcomes/what-we-do/health-surveys.html?gclid=CPj1nb6YoM8CFXQo0wodZXEDLQ	1	Health Status<br><br>8 domains (8 items)<br>Physical functioning (1)<br>Social functioning (1)<br>Role physical (1)<br>Bodily pain (1)<br>Mental health (1)<br>Role emotional (1)<br>Vitality (1)<br>General health (1)	Categorical: 2-6 options	Recall:<br>Standard 4-weeks<br>Acute 1-week	Requires algorithm to score domains<br><br>Norm-based scoring: score transformed to 0-100 (mean 50 (SD 10))<br><br>2 summary scales:<br>Physical Component Summary<br>Mental Component Summary	5-10 minutes<br><br>*Not reported in headache population*
Utility measures (3/6)						
EuroQoL EQ-5D (3L)<br><br>(EuroQoL Group, 1990)[47]<br><br>http://www.euroqol.org/<br><br>*Self-completion or interview administered*	3	Quality of Life<br><br>5 domains (5 items)<br>Mobility<br>Self-care<br>Usual activities<br>Pain/discomfort<br>Anxiety/depression	3-point descriptive response options: no problems, some problems, severe problems.	Today	Utility index value (society assigned value system algorithm): -0.59 to 1.00 where 1.00 is perfect quality of life, 0 is death, and <0 is a health state worse than death	2 to 5 mins<br><br>*Not reported in headache population*
Health Utility Index – 3<br><br>(HUI-3)<br><br>(Feeney et al, 2002)[48]<br><br>http://www.healthutilities.com/hui3.htm<br><br>*Self-completion or interview administered*	1	Multi-attribute health status classification system<br><br>Describes the comprehensive health state of an individual as 8 domains (attributes) (8 items):<br>Vision<br>Hearing<br>Speech<br>Ambulation<br>Dexterity<br>Emotion<br>Cognition	1 to 5 or 1 to 6 descriptive response options per attribute / domain, where 1 is best health, 5 or 6 is worst health.	Current	Standard algorithms.<br>0 to 1.00 where 1.00 is perfect QoL	5 mins<br><br>*Not reported in headache population*

		Pain				
Quality of Well-being Scale<br><br>(QWB)<br><br>(QWB - Kaplan et al, 1993 [49]; QWB-SA - Andresen et al, 1998 [50])<br><br>Interview and self-administered (SA)	1	Generic measure of HRQoL; used to calculate QALYs<br><br>QWB – interview administered *(fewer than QWB-SA)*<br>QWB-SA – self-administered (77 items)<br><br>Symptom scale<br>3 scales of function: mobility, physical activity, social activity		6-days<br>3-days	Overall score based on a preference-weighted average functioning in previous 6-days.<br><br>Utility index score: 0.0 (death) to 1.0 (perfect health)	QWB-SA: average 11 minutes

Appendix 5: Table 4: Characteristics of included studies (n= 46)

Study<br>(Author;yr)<br>[ref]	Country[g]<br>Language[g]	Population and Headache definition	Study: design, setting, sample size[g]	Mean [g]age (SD; range)	Gender distribution (F Female)[g]	Treatment descriptions[g]	% missing responses: acceptable?[g] *	PROM focus;<br>Additional info [g]
Andree et al 2010 [28]	Five countries: UK, Italy, Spain, Germany/Austria, France	Patients from mixed settings with diagnosis of headache: International Classification of Headache Disorders (ICHD-II) 2004	International cross-sectional survey:<br>Population recruitment:<br>UK and Italy: Headache/migraine associations;<br>France/ Austria/ Italy: Neuroscience clinics;<br>Germany/ Luxembourg: population based cohort;<br>Spain: GP population<br>Total n=426<br>UK n=131<br>Italy n= 60<br>Spain n=107<br>Germany/Austria n= 83<br>France n=45	Total:<br>44.0 (+/- 11.38)	Total:<br>F 75.0%	NA	Total: quoted as 66-100% (data NR) not quoted at item level	EUROLITE - development
Bagley et al 2012 [54]	Data from 9 countries: US, Canada, France, Germany, Spain, UK, Australia, Italy, Taiwan.	Chronic (CM) and Episodic Migraine (EM)<br>Detailed definitions *(p410)*	International Burden of Migraine Study (IBMS)<br>Web-based, cross-sectional population survey<br>Participants recruited from established database of headache / migraine patients (n	Total:<br>40.3 (11.4)<br>EM 40.2 (11.4)	Total:<br>F 83.5%<br>EM F 83.4%	N/A	Not reported at item level	MSQ 2.1<br>*Includes a 'review' of the development / earlier evaluation*

	Suggests that questionnaires completed in 'official language of the country' – but results then combined	EM: <15 HDPM<br><br>CM: >/= 15 HDPM	63,001): all received e-invitation to 'opt in' via web-link.<br><br>30.7% responded to email invitation; 55% eligible to participate.<br><br>Surveys completed by 81.9%<br><br>Total n 8726<br><br>EM n 8227<br><br>CM n 499	CM 41.7 (12.1)	CM F 85.6%			*papers for MSQ and HIT-6.*
Bigal et al 2003 [69]	US<br><br>US English	Patients registered with a specialist headache clinic<br><br>CM: daily or near-daily headaches lasting >4hrs if untreated, >15 days per month, fulfilling CDH.<br><br>EM: HIS for migraine +/- aura	Retrospective assessment of patient clinic notes (for those who had previously completed the MIDAS)<br><br>CM 182<br><br>EM 86	CM 38.3 (95% CI 36.5 to 40.1)<br><br>EM 36.1 (95% CI 34.1 to 38.0)	CM F 72.5%<br><br>EM F 68.6%	N/A	Not reported at group or item level	MIDAS
Bjorner et al 2003a [3]	US<br><br>US English	General population<br><br>At least 1 headache in 4/52 prior to interview (not hangover, cold, flu)	National Survey of Headache Impact (NSHI) – longitudinal survey (baseline and 3/12)<br><br>Sampling frame: randomly generated list of telephone numbers from 48 US states.	NR<br><br>*Reported elsewhere in NSHI papers*	NR	N/A	N/A	HIT development paper<br><br>Focus: item pool development. Informed by items from the: MSQ (v2), HDI, HIMQ, MIDAS

			Interviews with convenience sample of eligible respondents (mean duration 21.5mins (rge 17-27 mins) – schedule not detailed<br><br>Telephone interviews n= 1016<br><br>Headache prevalence in 4/52 period= 45.7%					
Blumenfeld et al 2010 [71]	Data from 9 countries: US, Canada, France, Germany, Spain, UK, Australia, Italy, Taiwan.<br><br>*Suggests that questionnaires completed in 'official language of the country' – but results then combined*	Chronic (CM) and Episodic Migraine (EM)<br><br>Detailed definitions (p410)<br><br>EM: <15 HDPM<br><br>CM: >/= 15 HDPM	International Burden of Migraine Study (IBMS)<br><br>Web-based, cross-sectional population survey<br><br>Participants recruited from established database of headache / migraine patients (n 63,001): all received e-invitation to 'opt in' via web-link.<br><br>30.7% responded to email invitation; 55% eligible to participate.<br><br>Surveys completed by 81.9%<br><br>Total n 8726<br><br>EM n 8227<br><br>CM n 499	Total:<br><br>40.3 (11.4)<br><br>EM 40.2 (11.4)<br><br>CM 41.7 (12.1)	Total:<br><br>F 83.5%<br><br>EM F 83.4%<br><br>CM F 85.6%	N/A	Not reported at item level	MIDAS<br><br>* Sub-division of Grade IV into IV-A severe disability (score 21-40) and IV-B very severe disability (41-270): reflects number of people with CM who fall into the grade IV category. *(ceiling effect – worst scores)*<br><br>*Includes a 'review' of the development / earlier evaluation papers for MSQ and HIT-6.*
Brown et al 2005 [79]	US	Care-seeking patients for migraine headache - registered at three sites representing varied	Cross-sectional survey questionnaire – primary focus evaluation of the HUI-3 in this population	44.0 (11.6)	F 87%	N/A	Not reported.	HUI-3<br><br>(MIDAS)

	US English	models of healthcare: primary care speciality clinic, non-profit HMO.<br><br>EM: physician diagnosed at least 1-year before study enrolment (medical chart review)	Consecutive patients recruited (each site total n50).<br><br>N 150		*(Causasian 87%)*			Mean HUI-3 scpre 0.62 (SD0.26)
Chatterton et al 2002 [40]	US<br><br>US English	Migraine diagnosis At least 1 migraine per month and	Cross-sectional survey questionnaire - primary focus evaluation of the M-TAQ; two-week test-retest (of individual items) on sub-set (n 100)<br><br>N 243	40.0<br><br>Range 18-63	F n 219 (91%)<br><br>*(Caucasian 63%)*	N/A	Not reported.	M-TAQ – development paper<br><br>(SF-36, MIDAS, Beck Depression Inventory)<br><br>Test-retest at 2-weeks (n 100)
Coeytaux et al 2006 [58]	US<br><br>US English	Specialist headache clinic<br><br>Chronic Daily Headache (CDH): presence of headache on >/= 15 days in the month prior to enrolling in clinical trial<br><br>Total recruited with CDH n 71	Randomized clinical trial: Usual medical care (UMC) (n37) v UMC plus acupuncture (n34)<br><br>Questionnaires administered at baseline and 6/52. Include patient self-report of meaningful improvement/no change/ deterioration at 6/52.	Mean 46.0;<br><br>range 19-83 yrs.	F 80% (n 57)<br><br>(93% white (n 66))	Usual medical care (UMC) (n37) v UMC plus acupuncture (n34) (consisting of 10 treatments over 6-wks)	Complete follow-up data for 71/74 enrolled patients (96%)	HIT-6<br><br>Patient-reported change in status at 6/52: 42% improved; 44% no change; 14% worse

		Mean duration of CDH 24.2 (SD 5.8) days						
Cole et al 2009 [60]	US and Canada<br><br>US English	1.Participants in clinical trial; and 2. Members of the general population<br><br>EM or Self-report Headache:<br><br>EM: minimum 6-mth history of Migraine (HIS criteria) with 3-12 Migraines per mth but not >15 HDPM during the 28-day prospective baseline period.<br><br>Self-report headache at least once in past 4-weeks (prior to phone interview)	Retrospective data analysis of two data-sets:<br><br>1. Pooled data from 2 randomized clinical trials (RCTs) (n 916) (Topiramite for Migraine prevention)<br><br>2. Population-based database (n 1016)	1. rge 12-65 yrs<br><br>2. rge 18-65 yrs<br><br>Additional data NR	NR	1. Double-blind placebo-controlled RCT<br><br>2. No intervention	Missing data: handling detailed p1181 (Bayesian multivariate imputation method)	MSQ v2.1<br><br>Focus on MID calculation for MSQ v2.1 (anchor and distribution based analysis) (p1182)
Cole et al 2007 [51]	US and Canada<br><br>US English	Participants in a clinical trial	Retrospective data analysis: pooled data from 2 randomized clinical trials (RCTs) (n 916) (Topiramite for Migraine prevention)	40.7 (10.7)	NR	Double-blind placebo-controlled RCT - Prophylactic migraine treatment	Missing data detailed.<br><br>MSQ v2.1: Baseline:	MSQ v2.1

		6-mth history of Migraine (IHS criteria) with 3-12 Migraines per mth but not >15 HDPM during the 28-day prospective baseline period.	MSQ v2.1 completed baseline, 2/4/6mths				range 0% (several items) to 0.44% (items 3 and 4)	Reports results for both 14-item (v2.1) and a revised 13-item measure (informed by results of initial testing)<br><br>IRT evaluation
Coon et al 2012 [38]	US<br><br>US English	Patients registered with specialist headache clinics (8 sites)<br><br>IHS diagnosis of migraine +/- aura for at least 1 year based on medical, medication and migraine history. During 3-mths prior to study enrolment – required to have x 3 to 8 migraine attacks per month and to have used triptans at least x2 per month.	Clinical study *(before / after)* (sumatriptan and naproxen versus usual therapy)<br><br>n 916<br><br>Baseline (BL) static CORS completed to reflect experience of 'usual therapy'.<br><br>After 2-mths treatment with Suma/Nap they completed the EOS static CORS (re-phrased to focus on Suma/Nap); comparative CORS also completed.	44.3 (11.0); range 19-65yrs	F 87.1%<br><br>*(91.2% white)*	Visit 1 (for 2-mths): participants treated any migraine with single-tablet formulation of Suma/Nap.	Not reported (at survey level)	Focus: treatment-specific measure -<br><br>CORS<br><br>Mean 22.4yrs (13.2; range 1-53) since Migraine onset)<br><br>Average HIT-6 at baseline 61.7 (range 42-76) suggesting severe impact of headache
Cramer et al 2001 [34]	US<br><br>US English	Patients with history of migraine.<br><br>Migraine not defined.	Development and initial evaluation of the HANA: data from three studies:	1.Mean NR. Range 19->65	1. F 804/994 (81%)<br><br>2. F 17/28 (61%)	1. N/A<br><br>2. Migraine prophylaxis clinical trial<br><br>2. Usual care	'No floor or ceiling effects' reported (but data not illustrated)	HANA - development paper<br><br>(HDI)

			1.Participants in the Life Impact Survey – a web-based survey (supported and widely publicised by various Headache and Migraine groups)<br><br>N 994<br><br>2.Participants in a migraine prophylaxis clinical trial.<br><br>N 28<br><br>3. 1-month test-retest cohort: 'no change in status' (anchor not reported)<br><br>N 25	2. 40.7 (11.1); range 16-69<br><br>3. 44.0; range 16-62	3. F 21/25 (84%)			
Davis et al 2002 [76]	Data from 6 countries: Spain, NZ, NL, Hungary, Finland, Canada	Clinical trial participants<br><br>Diagnosed according to IHS criteria<br><br>N 793	Multinational, open-label trial (oral naratriptan) vs 'usual therapy for M'. Duration 3-months<br><br>Baseline and 3-mths completion (end of trial)	Total population 38.4<br><br>Country mean age range: Spain 36.2 (9.1) to Canada 40.2 (8.7)	F 85%<br><br>*(98% white)*			PPMQ (original version)<br><br>Limited detail re PPMQ development (
Dodick et al 2007 [61]	US<br><br>US English	Patients aged >18 years with chronic migraine<br><br>Migraine defined according ICHD-II, with duration of 30 minutes or longer.	RCT – patients randomized 1 : 1 ratio to topiramate 100 mg/day or placebo (double-blind period 16weeks)<br><br>n328	38.2	F 85.3%	N/A<br><br>Report responsiveness (correlation of change scores)<br><br>Calculate MID (within-person (MIC)) for MSQv2.1	NR	MIDAS<br><br>MSQ v2.1<br><br>Subjective Global impression of change (SGIC)

		CM identified based on Silberstein–Lipton criteria, which required the presence of at least 15 headache days during the 28-day prospective baseline period				SGIC completed at the end of the study – 7-point scale (1= very much improved to 7= very much worse)<br><br>Physician GIC also completed.		
Dowson et al 2004 [39]	International study: 5 counties: UK, US, Spain, Germany, Italy.<br><br>All questionnaires translated into local language.	Patients registered at secondary care headache centres and attending for migraine treatment.<br><br>Migraine diagnosed according to IHS criteria (1998; 2004): participants aged 18-65yrs; minimum 1 yr history of migraine; average 1-4 attacks per month and minimum 24-hrs between attacks, and able to distinguish migraine from other headache.	Development and initial evaluation of the M-ACT: Open, prospective, multi-national, observational, two-visit study.<br><br>Baseline (n 185)<br><br>1-week test-retest (n 143) (no change in treatment during this time; but no health transition question reported).<br><br>Questionnaire completed in clinic or by telephone.	44.0; range 14-87 (93% aged 18-65)	F 68%<br><br>*(Caucasian 99.4%)*	N/A	NR	M-ACT – development paper<br><br>(SF-36, MIDAS, M-TAQ)
Gillard et al 2012 [73]	Data from 9 countries: US, Canada, France, Germany, Spain, UK, Australia, Italy, Taiwan.	Episodic and chronic M<br><br>Migraine defined according to International Classification of Headache Disorders (2nd Edition): plus	International Burden of Migraine Study (IBMS)<br><br>Cross-sectional, web-based observation study: paired observations of participants<br><br>Individuals randomly assigned to training or 'validation' samples	*NR in this paper for total pop.*<br><br>Presented by MSQ and HIT-6 completion (Table 1 p486)	Range 83-86% female	N/A	NR – assumed how dealt with??	EQ-5D with HIT-6 and MSQv2.1<br><br>**Conclusion:** relationship between the EQ-5D and both measures is adequate to use

	Suggests that questionnaires completed in 'official language of the country' – but results then combined	Chronic >/= 15 days per month; Episodic (< 15 HD per mth)<br><br>Total > 8500<br><br>CM >450<br><br>EM >8000	Aim: to develop empirical algorithms to estimate health state utility values from disease-specific QOL scores in individuals with migraine	Range (Median) 39-42 yrs (range 18-85)				regressions equations to estimate EQ-5D utility values. The preferred HIT-6 and MSQ algorithms can be used to estimate HSU in trials where a preference based measure is not used.
Kawata et al 2005 [55]	US<br><br>US English	New adult patients at university headache-speciality practice<br><br>Diagnosis not specified	Cross-sectional survey<br><br>All new patients who presented at clinic from Jan-Sept 2001<br><br>N 309	41.0 (SD 13)<br><br>Range 18-91 yrs.	F 77%	NR	Questionnaire response rate 309/369 (84%). HIT completion not reported at item level	HIT
Kilinster et al 2006 [74]	International study: 5 counties: UK, US, Spain, Germany, Italy.<br><br>All questionnaires translated into local language.	Patients registered at secondary care headache centres and attending for migraine treatment.<br><br>Migraine diagnosed according to IHS criteria (1998; 2004): participants aged 18-65yrs; minimum 1 yr history of migraine; average 1-4 attacks per month and minimum 24-hrs between attacks, and able to distinguish migraine from other headache.	Secondary analysis of data from the M-ACT study database (Dowson et al, 2000): evaluation of M-ACT reliability and validity.<br><br>Open, prospective, multi-national, observational, two-visit study. Data analysed for total population and per country<br><br>Baseline (n 185)<br><br>1-week test-retest (n 143) Questionnaire completed in clinic or by telephone.	44.0; range 14-87 (93% aged 18-65)	F 68%<br><br>*(Caucasian 99.4%)*	N/A	NR	M-ACT<br><br>(SF-36, MIDAS, M-TAQ)

Kimel et al 2008 [75]	US<br><br>US English	Minimum 6/12 history that met HIS criteria for Migraine with aura; experienced 2-6 Migraines per mth in the 3mths prior to screening (screening assessment to confirm diagnosis)	Data from two identical phase 3 trials: fixed dose sumatriptan + naproxen sodium Vs placebo<br><br>N 1304	40.1 (11.09)	F 87.8%	Fixed dose sumatriptan + naproxen sodium Vs placebo	Detailed p514-515	PPMQ-R<br><br>Evaluate psychometric properties in clinical trial setting
Kosinski et al 2003 [31]	US<br><br>US English	Members of the general population with experience of recent headache (self-diagnosed?)<br><br>A headache in the last 4-weeks that was not due to cold, flu, head injury, hangover	Two on-line community-based surveys (platform AOL's Opinion Place).<br><br>Time 1: n 1103<br><br>Time 2: n 540	Time 1. 37.0<br><br>Time 2. 37.5	Time 1: F 73%<br><br>Time 2: F 72%	N/A		(HIT and) HIT-6 development paper
Lipton et al 2003 [77]	US and UK<br><br>US / UK English	Migraine (n 399) and non-migraine (n 379) controls (data pooled for both populations p631)<br><br>HIS defined migraine (computer-assisted telephone interview (CATI)): HIS migraine +/- aura: 6 or more M in last year, but 15 or < headaches (any type) in previous month.	Two population-based studies (UK and US) – cross-sectional survey. Evaluation of migraine, quality of life (SF-12) and depression: patients with migraine versus non-migraine counterparts.	NR in this article – web-link provided.	NR	Nil.	NR	SF-12

Lipton et al 2009 [41]	6 countries: Canada, France, Germany, Italy, Spain, USA	Community-based population<br><br>IHS for Migraine +/- aura; at least x1 M per mth in the past 3mths; and no change in Rx for M in past 3mths.	Focus to establish the reliability and validity of a new measure – the M-TOQ: evaluation in five languages.<br><br>25 Primary care centres: N 253 (n50 per language)<br><br>Questionnaire completion during clinic-based interview: package of measures including M-TOQ, MIDAS, HIT-6, MSQoL. *(Unclear if all pen and paper or some touch-screen completion).*<br><br>Test-retest completion at 7-10 days (pen and paper completion).	43.1 (12.4)	F 90.1%	N/A<br><br>Evaluation of: data quality (missing values not reported), structural validity, internal consistency reliability, item-total correlation, convergent validity.	NR	M-TOQ<br><br>(HIT-6, MSQoL)
Lipton et al 2016 [59]	US<br><br>US English	Eligible adults (aged 18–65 years) - International Classification of Headache Disorders (ICHD-2) diagnostic criteria for CM .<br><br>To be eligible for inclusion, patients must have had >15 headache days during the 28-day screening period (baseline), during which >4 hours of each headache day were continuous headache and >50% were migraine or	Patients with CM from PREEMPT (Phase 3 REsearch Evaluating Migraine Prophylaxis Therapy) were randomized (1:1) to receive onabotulinumtoxinA or placebo for two 12-week cycles in the double-blind (DB) phase, followed by three 12-week cycles of open-label (OL) onabotulinumtoxinA (onabotulinumtoxinA/onabotulinumtoxinA (O/O) and placebo/onabotulinumtoxinA (P/O) groups, respectively).<br><br>n1236 participants (O/O, n607; P/O, n629) participated in both phases<br><br>HRQoL endpoints were assessed over 56 weeks using the HIT-6 and MSQv2.1	Baseline 41 (SD 10) yrs<br><br>White 90%	F 85%	PREEMPT clinical trial<br><br>Focus of report – the pooled HRQOL outcomes for 56-week treatment period.<br><br>Baseline<br>Mean HIT-6 65.4 (4.2)<br>Mean MSQ:<br>RR 38.6<br>RP 56.0<br>EF 42.2	Missing HIT-6 data were imputed using a prespecified, modified last observation carried forward technique (6,7). All observed MSQ data were analyzed without imputation for missing values.	HIT-6<br>36-49 no impact<br>55-55 substantial impact<br>60-78 severe impact<br><br>MSQv2.1<br>Range 0 (poor) to 100 (goodHRQoL)<br><br>*Paper describes where the measures have exceeded*

		probable migraine days.<br><br>Mean duration since CM onset 19yrs				At baseline most patients were severely debilitated by their migraines: 93% reporting a total HIT-6 score >60 (severe impact) and another 5% reporting a score of 56–59 (substantial impact)		*proposed MIC or MID*
Magnusson et al 2012 [32]	Canada<br><br>US English	Pre-IHS definition for chronic migraine; patients fulfilled criteria for transformed migraine +/- medication overuse<br><br>All patients were diagnosed with Migraine and had headache on >15 days per mth (fulfilling IHS criteria)	Patient registered with specialist headache centre studies:<br><br>1) Canadian Headache Outpatient Registry and Database (CHORD) (n 83);<br><br>2) Calgary Specialist headache clinic study (n 76)<br><br>Groups had similar demographics, number of H days and amount of H-related disability	1)41.0<br><br>2) 41.2	1)<br>F n64;<br>M n19<br><br>2)<br>F n64;<br>M n12	N/A	N/R	SF-36 Headache Modification (SF-36, HIT-6)<br><br>Group 1 completed the SF-36 Headache Modification; Group 2 the original SF-36 (v1)
Martin et al 2000 [36]	US<br><br>US English	Patients attending 4 outpatient headache speciality clinics<br><br>Diagnosed according to<br>IHS criteria	Multicenter, nondrug, prospective, parallel group, quasi-experimental design.<br><br>N= 267<br>(157 new and 110 stable)	New patients: 39.0<br><br>Stable patients: 44.6	F 90.6%	Stable patients: TAU<br><br>New patients: acute or prophylactic medications recommended by headache specialist	Item level: no missing observations or out of range values for either group	MSQ v2.1

Martin et al 2008 [65]	US<br><br>US English	Sub-set of participants with CH in a large multi-site RCT.<br><br>RCT inclusion: >21 yrs old; MIDAS score >5; intending to continue with general medical care; CH diagnosis defined below:<br><br>CH: tension-type, migraine or mixed aetiology.<br><br>Diagnosed by primary care physician (frequent and/or difficult to manage headaches)	Secondary analysis of RCT data: Headache Management Progammes (HMP): aimed at reducing H-related disability, improving process of care, reducing management costs.<br><br>Total n 124<br><br>Primary focus: MTSM evaluation in a sub-set of RCT population; Baseline (parts 1 and 2) and 6-mths (parts 3 and 4): self-completion (mail)	45.4 (11.6)	F 75%<br><br>*(59.7% Caucasian)*	N/A	NR	MTSM<br><br>(SF-36, MIDAS)
McKenna et al 1998 [37]	UK and US<br><br>UK and US English<br><br>Part of international study conducted in 8 countries.	Patients with history of migraine.<br><br>UK: Migraine diagnosed by clinician<br><br>US: Migraine diagnosed by clinical specialist<br><br>No further definition provided.	Development and initial evaluation of UK version.<br><br>1.Qualitative: interviews (UK n 30; US n25) and focus groups (US).<br><br>Recruitment: UK from general practice, British Migraine Association, pharma company employees.<br><br>US: from specialist clinics.<br><br>2. Postal survey (UK): Baseline n 87/90 completed questionnaire; 2-week test-retest questionnaire n87/87.	1. NR<br><br>2. 47.6; range 22-92	1.NR<br><br>2. F n72 (83%)	NA	NR	MSQoL – development paper

Niere & Quin 2009 [30]	Australia<br><br>Australian English	Patients attending private practice out-patient physiotherapy clinics (n45) for headache management<br><br>IHS diagnostic criteria (1988) for Migraine +/- aura, Migraine with aura and tension type headache.<br><br>Diagnostic criteria for cervicogenic headache.	Cross-sectional evaluation of the HDQ<br><br>Clinicians' selected consecutive patients meeting inclusion criteria<br><br>N 111	38.3 (12.2)<br><br>Range 18.0-74.0	F n93 (83.8%)	NA	Item response rates: 95%-100% (14 of 16 items at least 98%)	HDQ – development paper
Patrick et al 2000 [52]	8 countries: US, UK, France, Denmark, Germany, Italy, Spain, Sweden.	Previous participants in placebo-controlled trial: recruited into long-term observational study. Registered with specialist headache clinics.<br><br>IHS – diagnosed with Migraine	Non-comparative long-term observational study of zolmitriptan (Zomig) for acute treatment of migraine attack of any intensity (over 12-mths)<br><br>N 1383	41.2 (10.01); range 12-66	1190 F; 193 M (86:14)<br><br>Caucasian: 96.5%			Focus: MSQoL (20-item)<br><br>(Also SF-36 (US only; n= 1115).<br><br>Questionnaires completed: after treating 1, 5 and 17 M attacks or at 3-mthly intervals
Patrick et al 2003 [42]	US and UK English	Development and initial evaluation of new measure (MTSM)<br><br>Participants identified by their referring clinician as "a migraine patient starting a new treatment."	1.Participants in initial interviews / focus groups – item generation (US (30); UK (24)) and confirmation (23); Headache experts (US 3; UK 1). No additional detail.<br><br>2.Participants in clinic-based study (n=29) – to test the feasibility of using the MTSM in a clinical setting and to generate a preliminary data set from small group of patients.			New migraine treatment.<br><br>22 (75.9%) started on a triptan (ie, sumatriptan, naratriptan, rizatriptan, zolmitriptan, or almotriptan); 12/22 (54.5%) were already receiving a triptan, and the other 10	NR	Development and initial evaluation of the MTSM<br><br>Questionnaire completed baseline and follow-up.

				42.0 (11.3); range 24-64	28 F (97%)<br><br>Caucasian 25 (86%)	(45.5%) were receiving an analgesic.<br><br>Remaining 7 (24.1%) were changing migraine drugs; of these, 2 participants had been receiving a triptan: 1 was now starting an analgesic (naproxen) and 1 was starting an antidepressant (citalopram).<br><br>The 4 participants who were originally taking an analgesic started a new analgesic (naproxen or butalbital).<br><br>The remaining participant had been receiving an anxiolytic (alprazolam) and was now starting paroxetine (an antidepressant/anxiolytic also used for headaches).		Initial evidence for validity assessed against 24-hr MQoLQ and SF-36.
Pathak et al 2005 [33]	US and Germany	Migraine (IHS): at least 3 migraines in previous 12mths.	Stages in the development of the FAIM – a new measure.<br><br>1) Focus groups in US and German – limited detail<br>2) Pre-testing – item evaluation and reduction: n153 US and n148 Germany<br>3) Pilot test and final item reduction: n75 US and n83 Germany	Study 2: US 37.5 (13.2)<br><br>G 43.3 (13.7)<br><br>Study 3: US 38.8 (11.2)<br>G 41.0 (13.5)	F<br><br>Study 2:<br><br>US 66.2%<br><br>G 71.5%<br><br>Study 3:<br><br>US 76.8 %<br><br>G 69.9%	Stages in PROM development. No treatment.	Detailed per stage of developmet	Development of the FAIM
Rendas-Baum et al 2013 [12]	US, Canada, UK, Croatia, Germany and Switzerland.	International trial participants with diagnosis of migraine:	Secondary analysis of data from two multicentre double blind placebo controlled RCTs of chronic migraine patients receiving BOTOX as prophylaxis	Study 1:<br><br>41.6 (10.5)	Trial 1:<br><br>F 87.5%		Not reported	Comparative evaluation of the MSQ v2.1 and HIT-6

		ICHD-II for migraine, with the exception of "complicated migraine"<br><br>≥15 headache days during 4-week baseline phase; each headache day of ≥4 hours of continuous headache;<br><br>≥50% of baseline headache days migraine/probable migraine days.	Total N 1376<br><br>Trial 1: n 672<br><br>Trial 2: n 704	Study 2:<br><br>41.0 (10.6)	Trial 2:<br><br>F 85.4%			
Rendas-Baum et al 2014 [53]	US, Canada, UK, Croatia, Germany and Switzerland.	International trial participants with diagnosis of migraine:<br><br>ICHD-II for migraine, with the exception of "complicated migraine"<br><br>≥15 headache days during 4-week baseline phase; each headache day of ≥4 hours of continuous headache;<br><br>≥50% of baseline headache days migraine/probable migraine days.	Secondary analysis of data from two multicentre double blind placebo controlled RCTs of chronic migraine patients receiving BOTOX as prophylaxis<br><br>Total N 1376<br><br>Trial 1: n 672<br><br>Trial 2: n 704	Study 1:<br><br>41.6 (10.5)<br><br>Study 2:<br><br>41.0 (10.6)	Trial 1:<br><br>F 87.5%<br><br>Trial 2:<br><br>F 85.4%		Not reported	Comparative evaluation of the HIT-6 and MSQ v2.1
Revicki et al 2006 [43]	US<br><br>US English	Primary Care and neurology speciality clinics (n=50)	Longitudinal observational study: patients receive usual medical care; study investigators had discretion to change or prescribe medications	39.0 (11.0)	F n 181 (91%)		Detailed Table 3 (p 246)	PPMQ-R<br><br>Development / revision paper

		Documented diagnosis of migraine +/- aura (1988 IHS criteria 1.1 and 1.2): 2 to 8 Migraine attacks per month for at least 3-mths prior to study enrolment.<br><br>Able to distinguish Migraine from other Headache	N 200<br><br>Convenience sampling from participating clinics (not detailed)		*(Caucasian n 63 (82%))*		Ceiling effects for all 10 Bothersome items (>50%) and two ease of use items.	Participants kept a Migriane diary and completed the draft PPMQ-R at 24-hrs post-Rx for each M attack<br><br>(MSQv2.1)
Sauro et al 2010 [70]	Canada<br><br>Canadian English	Patients registered with Neurology Outpatient practices - patients referred by family physician or other specialist<br><br>Patients diagnosed according to IHS criteria - except patients with chronic daily headache (headache on 15 days a month or more).	Cross-sectional evaluation<br><br>Patients identified from the Canadian Headache Outpatient Registry and Database (CHORD) – patients registered with five neurology outpatient practices (Sept 2001-Jan 2004)<br><br>N 798	40.3 (SD 13.7)	F 77%	NR	Response rate 92%<br><br>Item level: only those with 'valid scores' included in the analysis.	HIT-6
Sieber et al 2000 [64]	Canada<br><br>Canadian English	n89 adults 'known to suffer with migraine'	Cross-sectional, comparative evaluation of QWB and QWB-SA for patients with migraine.<br><br>Postal self-completion of QWB-SA<br><br>Telephone-administered completion of QWB	42.2 (9.8)<br><br>Range 36 to 64yrs	F 87%	Questionnaires completed at 3-points: first, on a day when migraine had not been experienced within previous 7-days; 2nd and 3rd within 48hrs of onset of migraine.	Not reported.<br><br>Greater number of completions of QWB than QWB-SA	QWB (interview)<br><br>QWB-SA (self-administered)

Stafford et al 2012 [72]	UK<br><br>English	Members of the general population who had recently experienced a Migraine:<br><br>IHS definition: Migraine +/- aura.<br><br>At least 1 Migraine in the last 7 days and history of physician diagnosed Migraine for at least 6mths.	Cross-sectional observational study.<br><br>Recruited via Migraine support groups and support group databases – eligibility confirmed via telephone interview. Aged >18yrs.<br><br>N 105<br><br>Mean number of monthly Migraines 5.22 (4.1); range 1 to 20.<br><br>MIDAS grade: iv 51/106<br><br>Iii 32/106<br><br>Ii 14/106<br><br>I 9/106	47.45 (11.71)	F n 81 (76.4%)<br><br>*(Caucasian n 89 (83.2%))*		Missing items dealt with as recommended by EQ-5D developers<br><br>1 patient did not complete EQ-5D for current health status – therefore numbers reduced from 106 to 105	EQ-5D (3D)<br><br>Focus: generating utilities to reflect Migraine severity for participants most recent attack (past 4-weeks) and their current health state outside of an attack.<br><br>Tension-related aspects of M not evaluated.<br><br>EQ-5D self-completed retrospectively to reflect most recent M: completed for each level of M severity experienced (mild, mod and/or severe) during this attack. Also completed EQ-5D to reflect current non-M health state.
Stewart et al 1999 [35]	US and UK<br><br>US and UK English	Computer-Assisted Telephone Interview (CATI) based diagnosis of migraine (IHS criteria)	Postal self-completion of MIDAS – population based samples of patients with migraine-headache confirmed with CATI: UK n100 and US n97<br><br>Baseline and test-retest completion at 18-days	Range 18-55yrs	F<br><br>US 83.5%<br><br>UK 60%	N/A<br><br>Evaluation of MIDAS reliability.	NR	Initial development / refinement of MIDAS and testing of reliability

Stewart et al 1999 [66]	US<br><br>US English	Computer-Assisted Telephone Interview (CATI) based diagnosis of migraine (IHS criteria)	Postal self-completion of MIDAS – population based samples of patients with migraine-headache confirmed with CATI: US n97 and n80 non-migraine subjects<br><br>Baseline and test-retest completion at 21-days	Range 18-55yrs	F<br><br>US 83.5%	N/A<br><br>Evaluation of MIDAS reliability	NR	Initial reliability resting of MIDAS
Stewart et al 2000 [67]	US<br><br>US English	Patients with a confirmed diagnosis of migraine<br><br>Initial migraine status determined from IHS based CATI algorithm<br><br>Migraine status confirmed by clinical examination at initial clinic visit	Population based interview survey<br><br>n=144<br><br>*(a total of 12967 diary days)*	37.6 (9.3)	F 75.7%	NA (Treatment As Usual)	Interview participation rate 67%<br><br>Inadequate diary entry 16.5%	MIDAS
Stewart et al 2003 [68]	US and UK<br><br>English	Total n= 397<br><br>59% MIDAS grade III or IV<br><br>Initial migraine status determined from IHS based CATI algorithm	Secondary data analysis from three population based studies	Mean NR<br><br>62% aged 25 – 44yrs<br><br>(range <25 to 55+)	F 78%	N/A	NR	Focus: relationship between headache features (freq, pain intensity, quality, assoc symptoms) and the MIDAS<br><br>Telephone interview-based completion??

Turner-Bowker et al 2003 [7]	US<br><br>US English	General population<br><br>Patient self-report or patient-reported doctors' diagnosis of migraine	Cross-sectional Internet (AOL) and mail population survey<br><br>Convenience population sample<br><br>Total N=7557<br><br>Migraine n=1478	Range 35–44 years	F 53%	Not assessed	Total questionnaire response rate 27.8%<br><br>Internet response rate unreported	SF-8
Vernon & Lawson 2015 [29]	Canada/UK<br><br>Canadian English	Patients attending a chiropractic clinic and self-diagnosed with primary migraine, tension-type or cervicogenic headache	Cross-sectional completion of HADLI<br><br>Participants recruited by advertisement/ personal solicitation<br><br>N 53	37.3 (12) yrs.	F 41 (77.4%)	NA	NR	HADLI development paper
Ware et al 2003 [57]	US<br><br>US English	General population<br><br>At least 1 headache in 4/52 prior to interview (not hangover, cold, flu)	Two studies:<br><br>1.National Survey of Headache Impact (NSHI) – longitudinal survey (baseline and 3/12)<br><br>Sampling frame: randomly generated list of telephone numbers from 48 US states.<br><br>Telephone interviews with convenience sample of eligible respondents n= 1016<br><br>Headache prevalence in 4/52 period= 45.7%<br><br>2.On-line community-based survey (platform AOL's Opinion Place): respondents randomly			Detail: Ware 2000 *Med Care) and Bjorner 2003a [9]		HIT (total)<br><br>CAT-HIT<br><br>HIT-6-D ('static') and improved version (HIT-6) in study 2<br><br>SF-8<br><br>Focus: evaluation of CAT-based estimates vs 'static' scores: reliability and validity (plus

			selected and screened for eligibility: n 1103 included.<br><br>Test-retest at 2-weeks for sub-group (n 540).					respondent burden)
Xu et al 2011 [78]**	US<br><br>US English	Participants in a multi-centre clinical trial.<br><br>M defined by IHS (+/- aura); Between 1 – 6 moderate to severe Migraine attacks per month	Secondary analysis of data from a multi-centre (20 sites) double-blind RCT of treatment for acute Migraine (Talcagepant). Rx duration was for a single Migraine attack of moderate to severe intensity.<br><br>N 330<br><br>All data pooled for EQ-5D analysis. Disutilities calculated for selected patients with mod/severe Migraine pain at baseline who reported pain freedom at 24-hrs (difference in EQ-5D scores between time-points calculated)	Mean NR<br><br>Range 20-65 yrs.	F n 292 (88.5%)<br><br>*(Caucasian n 259 (78.5%))*	.	NR	EQ-5D (3D)<br><br>Completed at baseline (whilst experiencing a mod/severe M and prior to dosing) and 24-hr post Rx within an acute M attack. Patients also completed Pain levels during this time (4-grade: no/mild/mod/ severe).
Yang et al 2010 [56]	US<br><br>US English	Population based survey – self-completed questionnaires<br><br>CM and EM: Categorised into 3-groups:<br><br>1) CM (=/>15 HDPM) = 6.4%;2) EM (<15 HDPM) = 42.1%; 3)	Participants in National Survey of Headache impact (NSHI) (n= 1096) and the HIT-6 Validation study (n= 54)<br><br>N= 2049<br><br>Focus of analysis: reliability and validity of HIT-6 in patients with varying headache frequency days.	Mean NR<br><br>Range 18-69yrs<br><br>56.3% aged 18-39	F 75%	NA	NR	HIT-6<br><br>(SF-8)<br><br>HIT-6 scores calculated across the 3 groups:<br>1) CM = 62.5 (7.8);<br>2) EM = 60.2 (6.8);<br>3) Non-M headache = 49.1 (8.7)

		Non-M headache = 51.5%<br><br>Survey included a Migraine screener ID Migraine criteria – p359) and number of headache days per month (HDPM)						
**Study**<br><br>**(Author;yr)**<br><br>**[ref]**	**Country[g]**<br><br>**Language[g]**	**Population and Headache definition**	**Study: design, setting, sample size[g]**	**Mean [g]age (SD; range)**	**Gender distribution (F Female)[g]**	**Treatment descriptions[g]**	**% missing responses: acceptable?[g] ***	**PROM focus;**<br><br>**Additional info [g]**

Footnote:

- Missing values: a. At survey level; b. At item level (data quality – frequency with which items were missing (ie. Non-completed) and how did the authors deal with missing values?

Bagley 2012: HDPM Headache days per month (p410 for detail); IBMS – International Burden of Migraine Study (2009).

Appendix 6: Table 5 Content comparison at item level (number of items) of condition-specific measures (n= 17)

**PROM**[a]	**Items (n)** [b]	**Domains of Health-related Quality of Life (Ferrans et al, 2005 [25])***												**Response to Treatment**			
		Symptom Status				***Functional Status***							***General Health Perception***				
		**Symptoms**				**Physical**	**Social / Role**				**Psychological**						
							**Social / Role**		**ADL / Work**		**EWB**	**Cognition**		**Return to normal**	**Confidence / Satisfaction with Rx**	**Side effects**	**Other**
		**General**	**Headache Frequency**	**Headache Intensity**	**Pain**	**Physical**	**Limit**	**Prevent**	**Limit**	**Prevent**							
Condition-specific (17)																	
Migraine impact (5/17)																	
FAIM [33]	9 + 5						5				5		4				
HANA [34]	7	1					1		1		3		1				
MIDAS [35]	5 + 2		1*	1*				1	4								
MSQv2.1[36]	14	2					2	1	3	2	3	1					
MSQoL [37]	20						6		10		4						
Headache impact (6/17)																	
EUROLIGHT [d] [28]	103	X	X	X	X	X	X		X		X	X					
HADLI [29]	9	1				1		2	4			1					
HDQ [30]	9				2		1		3	3							
CAT-HIT [e] [3,57]	54	X			X		X		X		X	X					
HIT-6 [31]	6	1			1		1		1		1	1					
SF-36 Headache [32]	6/36				2	1	2		1								
Response to migraine-specific treatment (6/17)																	
Static CORS<br>Comp CORS<br>[38]	24<br><br>8	9<br><br>4			5				2		3	1		1<br><br>1	3<br><br>3		
M-ACT [39]	27<br>4													20<br>2	7<br>2		
M-TAQ [40]	9	1	1							1				1	1		4
M-TOQ15<br>M-TOQ-5<br>[41]	15<br>5	1		1	2<br>1				2<br>1		1			1	4<br>2	3<br>1	
MTSM [42]	45	1			3				1						3		
PPMQ-R [43]	19<br>10	3<br>8			6		2		2		1<br>1	 1	2 (cost)		3		

Footnote:

****Ferrans et al (2005) revision to the Wilson and Cleary Model of Health Related Quality of Life (HRQOL)[25]:*** The model describes five levels of patient outcomes from biological and psychological variables through to overall quality of life (subjective well-being assessed by an individuals perceived level of happiness or satisfaction with life), and includes symptoms (for example, physical, emotional, cognitive symptoms perceived by the patient), functional status (for example, physical, social, role and psychological function) and general health perceptions (a subjective rating incorporating all of the preceding health concepts). Additionally, characteristics of the individual (such as values and preferences) and those of the environment (such as social, economic and psychological support) are considered.

[a] PROM content; [b] Number of items per PROM

Migraine-impact:

[a] FAIM – Functional Assessment in Migraine: 9 items across three domains. 1) Attention/Thought (5 items); 2) Perception (4 items); 3) Activity and Participation (5 items)

HANA - Headache Needs Assessment: 7 items to reflect migraine frequency and bothersomeness. 1 item in each of following area: Anxiety/worry; depression/discouragement; self-control; energy; function/work; family/social activities; overall impact of migraines.

MIDAS - Migraine Disability Assessment Score: 3 domains (5 scored items): Missed days/ reduced productivity at paid work (2 items); Missed days/ reduced productivity at household work (2 items); Missed non-work activities (1 items)

MSQv2.1 - Migraine-Specific Quality of Life Questionnaire version 2.1: 3 domains (14 items): 1) Role function – Restrictive (RR)(7 items): social – family/friends; leisure (2); work/ADL (3); cognition (1); symptoms: fatigue (1); 2) Role function – Preventive (RP)(4 items): symptoms: fatigue (1); work /ADL (2); social (1); 3) Emotional function (EF)(3 items): frustration (1); feeling a burden (1); letting others down (1).

MSQOL - Migraine-Specific Quality of life: 3 domains (20 items): 1) Avoidance behaviours (10); 2) Social relations (6); 3) Feelings (4).

Headache impact:

EUROQLIGHT: 6 sections (103 items): 1) Biographical (age, gender, language and employment); 2) Screening questions for headache; 3) Diagnostic questions; 4) Questions about any headache experienced 'yesterday' (point prevalence); 5) Use of healthcare resources (medicines, investigations, consultations, etc.); 6) Impact of headache on work, family life and social activities: includes items taken from the WHOQOL (8-items), the HALT-index and HADS. [d] *EUROLIGHT – number of items per domain not clear (denoted by X).*

HADLI - Headache Activities of Daily Living Index: 1 domain (9 items): Personal care, Lifting, Reading (including computers), Sleeping (over last week), Exercising (over last week), Social activities, Work, Driving or travelling, Recreation.

HDQ – Headache Disability Questionnaire: 3 domains (9 items): 1) Pain (2 items): usual pain intensity; when pain is severe; 2) Activity Limitation (4 items): Decreased efficiency in non-work activities; Decreased ability to work/study; Decreased efficiency in housework or chores; Proportion of times when work is missed; 3) Activity Prevention (3 items): Number of days where chores prevented;
Number of days non-work activities prevented; Number of days in last month when had to lie down for >1 hour.

HIT (CAT-HIT) - Headache Impact Test: One domain 'Headache Impact': 54-item 'item bank'. Items cover a wide spectrum of headache impact, including minor headache. Items cover of pain, role and social functioning, energy/fatigue, cognitive function, and mental health.

[e] CAT-HIT: Number of completed items is individualised and determined by 'stopping rule': mean number 6/54 items (denoted by X)

HIT-6 – Headache Impact Test -item (static): 6 domains (6 items): 1) Headache pain severity(1); 2) Social functioning - u*sual daily activities*(1); 3) Role functioning *(how often do you wish you could lie down?)* (1); 4) Vitality(1); 5) Cognitive functioning(1); 6) Psychological distress(1).

SF-36 Headache - Inserts 'including your headaches' to 6/36 items: physical functioning (item 3); role limitation - physical (item 4); social functioning (items 6 and 10); bodily pain (items 7 and 8)

Response to migraine-specific treatment:

CORS - Completeness of Response to Migraine Therapy Survey: Two modules:1.'Static CORS' – 5 domains (24 items): 24-items address: Symptoms, Emotional well-being, Cognition, ADL, Function, Confidence in current medical. 2. 'Comparative CORS (8 domains (8 items)): Completeness of Relief (1), Speed of Relief (1), Persistence of Relief (1), Return to Normal Function (1), Fatigue (1)
Confidence in Treatment (2), Overall Satisfaction (1) – most satisfied.

M-ACT - Migraine – Assessment of Current Therapy: 4 domains (27 long-form/ 4 short): Headache impact (11/1); Global assessment of relief (9/1); Consistency of response (3/1); Emotional response (4/1).

M-TAQ - Migraine Therapy Assessment Questionnaire: 9 items - Migraine control, frequency of attacks, knowledge and behavioural barriers, economic burden, treatment satisfaction.

M-TOQ - Migraine-Treatment Optimization Questionnaire M-TOQ: 15- and 5-item versions: 5 domains (15/5 items): Functioning (3/1), Rapid relief of headache (3/1), Consistency of response (3/1), Prevention of recurrence (3/1), Side effects (3/1).

MTSM - Migraine Treatment Satisfaction Measure: 4-part assessment: 1) Expectations of Treatment for Migraine (TE-M; 9 items); 2) Importance Ranking for Migraine Treatment (IR-M; 9 items); 3) Outcomes of Treatment for Migraine (TO-M; 9-items); 4) Satisfaction with Migraine Treatment (PST-M; 9-items). Considered across 9 attributes ('items') associated with migraine relief: Pain relief; Speed of relief; Freedom from pain; Additional symptoms; Confidence in treatment; Disruption in life; Dosing; Freedom from relapse; Ease of use

PPMQ-R - Patient Perception of Migraine Questionnaire -Revised: Core: 4 domains (19 items): Efficacy: satisfaction with treatment efficacy (11); Function: ability to perform usual activities (4); Ease of Use (2); Cost (2) (*may be removed where 'cost' is not a consideration*). Additional fifth domain (10 items): 'Bothersomeness' or 'Tolerability' - related to side effects (10). 3 global items: Overall satisfaction with medication effectiveness; Side effects; General treatment.

Appendix 7: Table 6: Methodological quality (COSMIN[a]) of each study (n=46) per PROM (n=23) and investigated measurement property.

**PROM[b] Study (n)**	**Country (language)**	**Headache definition[c]**	**(n)**	**Reliability**			**Validity**				**Responsiveness**		**Interpretability**
				**Internal consistency**	**Reliability**	**Measurement error**	**Content validity**	**Structural validity**	**Hypothesis testing**	***Known-groups validity***	**Responsiveness (COSMIN)**	**Responsiveness - other**	
Condition-specific (17)													
Migraine-impact (5/17)													
**FAIM (1)**													
Pathak et al 2005 [33]	US English German	M	69 83	Good			Fair	Good	Good				
**HANA (1)**													
Cramer et al 2000 [34]	US English	M		Poor	Poor		Poor	Poor	Poor	*Poor*			
**MIDAS (12)**													
Stewart et al 1999a [35]	US and UK English	M (IHS)	US 97 UK 100	Poor	Poor								
Stewart 1999b [66]	US English	M (IHS)	97	Poor	Poor								
Stewart et al 2000 [67]	US English	M (IHS)	144						Fair				
Stewart et al 2003 [68]	US and UK English	M (IHS)	397						Poor				
Bigal et al 2003 [69]	US English	CM	182							*Fair*			
Dodick et al 2007 [61]	US English	CM (ICHD-II)	328								Fair		
Martin et al 2008 [65]	US English	CH – TT/M/mixed	124						Fair				
Lipton et al 2009 [41]	Multiple	M (IHS)	253	Poor					Fair				
Sauro et al 2010 [70]	Canadian English	CH (IHS)	798						Fair	*Fair*			
Blumenfeld et al 2010 [71]	Multiple	CM; EM	8726							*Fair*			
Yang et al 2010 [56]	US English	CM; EM	>600						Good	*Good*			
Bagley et al 2012 [54]	Multiple	CM; EM	8726 total						Fair				
Stafford et al 2012 [72]	UK English	M (IHS)	105						Fair				
**MSQ v2.1 (9)**													
Martin et al 2000 [36]	US English	M (IHS) / EM	267	Good	Good		Fair		Excellent	*Excellent*			

Revicki et al 2006 [43]	US English	EM (IHS 1.1, 1.2)	200						Good				
Dodick et al 2007 [61]	US English	CM (ICHD-II)	328								Fair		Poor
Cole et al 2007 [51]	US English	EM (IHS)	916	Excellent				Excellent	Fair				
Cole et al 2009 [60]	US English	EM											Fair
Blumenfeld et al 2010 [71]	US English	CM and EM								*Fair*			
Bagley et al 2012 [54]	US English	CM; EM	8726 total	Fair					Fair	*Fair*			
Gillard et al 2012 [73]	Multiple	CM; EM	8726							*Fair*			
Rendas-Baum et al 2013 [12]	US English	CM (ICHD-II)	1376	Good	Good			Good	Good	*Good*		*SRM*	
Rendas-Baum et al 2014 [53]	US English	CM (ICHD-II)	1376						Good				
Lipton et al 2016 [59]	US English	CM (ICHD-II)	1236									*% exceeding MIC or MID*	
**MSQoL (3)**													
McKenna et al 1998 [37]	UK English	M (IHS)		Poor	Fair		Excellent		Poor	*Fair*			
Patrick et al 2000 [52]	US English	M	1376	Fair			Fair	Good		*Fair*		*ES; SRM*	Poor
Lipton et al 2009 [41]	Multiple	M (IHS) (EM?)	253	Poor					Fair				
Headache-impact (6/17)													
**EUROLIGHT (1)**													
Andree et al 2010 [28]	Multiple UK English	Headache – all types	426 131	Poor	Poor		Fair		Poor	*Poor*			
**HADLI (1)**													
Vernon et al 2015 [29]	Canadian English	Self-diagnosed headache: M, TT, CG	53	Fair			Poor	Fair					
**HDQ (1)**													
Niere & Quin 2009 [30]	Australian	M (IHSD); Mixed: TT, CG, 'other'	111	Fair			Poor	Fair					

**HIT (3)**													
Bjorner et al 2003a [3]	US English	H (C or E)	1016				Fair	Excellent					
Ware et al 2003 [57]	US English	H	1.1016<br>2.1103		Fair		Fair		Good	*Good*	Poor		
Kosinski et al 2003a [31]	US English	H	1103		Fair		Fair		Fair	*Fair*			
**HIT-6 (12)**													
Kosinski et al 2003a [31]	US English	H	1103	Fair	Fair		Fair		Fair	*Fair*	*Fair*		
Ware et al 2003 [57]	US English	H	1.1016<br>2.1103		Fair		Fair		Good	*Good*	Poor		
Kawata et al 2005 [55]	US English	H	309	Fair				Fair	Fair				
Coeytaux et al 2006 [58]	US English	CDH	71										Fair
Lipton et al 2009 [41]	Multiple	M (IHS) (EM?)	253	Poor					Fair				
Sauro et al 2010 [70]	Canadian English	CH (IHS)	798						Fair	*Fair*			
Yang et al 2010 [56]	US English	CM; EM	>600	Good	Fair				Good+	*Good*			
Gillard et al 2012 [73]	Multiple	M (ICHD): CM and EM	9048							*Fair*			
Bagley et al 2012 [54]	US English	CM; EM	8726 total						Fair				
Magnusson et al 2012 [32]	US English	CM (+/- MOU)	159						Poor				
Rendas-Baum et al 2013 [12]	US English	CM (ICHD-II)	1376						Good				
Rendas-Baum et al 2014 [53]	US English	CM (ICHD-II)	1376	Good	Good			Good	Good	*Good*		*SRM*	
Lipton et al 2016 [59]	US English	CM (ICHD-II)	1236									*% exceeding MIC or MID*	
SF-36 'Headache-specific' (1)													
Magnusson et al 2012 [32]	US English	CM	159				Poor / nil		Fair				
Response to migraine-specific treatment (6/17)													

**CORS (1)**													
Coon et al 2012 [38]	US English	M (IHS)	916	Fair			Good	Fair	Fair		Fair		
**M-ACT (2)**													
Dowson et al 2004 [39]	Multiple	M (IHS)	185		Fair		Poor		Poor				
Kilinster et al 2006 [74]	Multiple	M (IHS)	185		Fair				Poor				
**M-TAQ (1)**													
Chatterton et al 2002 [40]	US English	M (IHS)	243		Fair		Fair		Fair				
**M-TOQ 19/15/5 (1)**													
Lipton et al 2009 [41]	Multiple	M (IHS) (EM?)	253	Fair	Fair		Fair	Fair	Fair				
**MTSM (2)**													
Patrick et al 2003 [42]	US English	M	29	Poor			Good		Fair	*Fair*			
Martin et al 2008 [65]	US English	CH – TT/M/mixed	124	Fair			Poor	Fair	Good	*Good*			
**PPMQ-R (2)**													
Revicki et al 2006 [43]	US English	EM (IHS 1.1, 1.2)	200	Excellent	Fair		Excellent	Excellent	Excellent	*Good*		*ES*	Fair
Kimel et al 2008 [75]	US English	M (IHS)	1304	Excellent				Good	Good	*Good*			
Generic measures (6)													
Profile measures (3/6)													
**SF-36 (5)**													
Patrick et al 2000 [52]	US English	M	1376									*ES, SRM*	
Martin et al 2000 [36]	US English	M (IHS) / EM	267						Good				
Davis et al 2002 [76]	Multiple	M (IHS)	793						Poor				
Kawata et al 2005 [55]	US English	H	309						Fair				
Martin et al 2008 [65]	US English	CH – TT/M/mixed	124						Fair				
**SF-12 (1)**													
Lipton et al 2003 [75]	US and UK English	M (IHS)	M 399							*Fair*			

			Control 379										
**SF-8 (4)**													
Kosinski et al 2003a [31]	US English	M (self-report)	1103						Fair				
Ware et al 2003 [57]	US English	H	1.1016<br>2.1103						Good				
Turner-Bowker et al 2003 [7]	US English	M (self-report)	465						Good	*Good*			
Yang et al 2010 [56]	US English	CM; EM	>600						Good	*Good*			
Utility measures (3/6)													
**EuroQoL EQ-5D-3L (3)**													
Xu et al 2011 [78]**	US and UK English	M (HIS)	330							*Poor*			
Gillard et al 2012 [73]	Multiple	M (ICHD): CM and EM	9715<br>CM<br>555<br>EM<br>9160							*Fair*			
Stafford et al 2012 [72] *	Uk English	M (IHS)	105						Fair	*Fair*			
**HUI-3 (1)**													
Brown et al 2008 [79]	US English	EM	150							*Poor*			
**QWB and QWB-SA (1)**													
Sieber et al 2000 [64]	US English	M	89		Poor					*Fair*			

Footnote:

[a] COSMIN – Consensus on Standards for Measurement Instruments. Four-grade rating for study methodological quality: Excellent, Good, Fair Poor. [20,21]

[b] PROM acronyms (detailed in text and Tables 1 and 2)

[c] Headache definition: H – Headache (general); CH – Chronic Headache; TT – Tension Type; CG – Cervico Genic; CDH – Chronic Daily Headache; M – Migraine; EM – Episodic M; CM – Chronic M; IHS – International Headache Society – headache classification system (https://www.ichd-3.org/); ICHD – International Classification of Headache Disability (https://www.ichd-3.org/).

*Focus of paper: generating utilities to reflect Migraine severity; **Focus of paper: calculating disutilities.
